# Chromatin-enriched RNAs mark active and repressive *cis*-regulation: An analysis of nuclear RNA-seq

**DOI:** 10.1371/journal.pcbi.1007119

**Published:** 2020-02-10

**Authors:** Xiangying Sun, Zhezhen Wang, Johnathon M. Hall, Carlos Perez-Cervantes, Alexander J. Ruthenburg, Ivan P. Moskowitz, Michael Gribskov, Xinan H. Yang

**Affiliations:** 1 Department of Pediatrics, The University of Chicago, Chicago, Illinois, United States of America; 2 Department of Biological Sciences, Purdue University, West Lafayette, Indiana, United States of America; 3 Department of Molecular Genetics and Cell Biology, The University of Chicago, Chicago, Illinois, United States of America; 4 Department of Computer Science, Purdue University, West Lafayette, Indiana, United States of America; La Jolla Institute for Allergy and Immunology, UNITED STATES

## Abstract

Long noncoding RNAs (**lncRNAs**) localize in the cell nucleus and influence gene expression through a variety of molecular mechanisms. Chromatin-enriched RNAs (**cheRNAs**) are a unique class of lncRNAs that are tightly bound to chromatin and putatively function to locally *cis*-activate gene transcription. CheRNAs can be identified by biochemical fractionation of nuclear RNA followed by RNA sequencing, but until now, a rigorous analytic pipeline for nuclear RNA-seq has been lacking. In this study, we survey four computational strategies for nuclear RNA-seq data analysis and develop a new pipeline, Tuxedo-ch, which outperforms other approaches. Tuxedo-ch assembles a more complete transcriptome and identifies cheRNA with higher accuracy than other approaches. We used Tuxedo-ch to analyze benchmark datasets of K562 cells and further characterize the genomic features of intergenic cheRNA (**icheRNA**) and their similarity to enhancer RNAs (**eRNAs**). We quantify the transcriptional correlation of icheRNA and adjacent genes and show that icheRNA is more positively associated with neighboring gene expression than eRNA or cap analysis of gene expression (**CAGE**) signals. We also explore two novel genomic associations of cheRNA, which indicate that cheRNAs may function to promote or repress gene expression in a context-dependent manner. IcheRNA loci with significant levels of H3K9me3 modifications are associated with active enhancers, consistent with the hypothesis that enhancers are derived from ancient mobile elements. In contrast, antisense cheRNA (**as-cheRNA**) may play a role in local gene repression, possibly through local RNA:DNA:DNA triple-helix formation.

## Author summary

Nuclear RNA-seq provides a powerful way to profile the transcriptional landscape, especially the noncoding transcriptome. Through analyzing nuclear RNA-seq, the chromatin-enriched RNA (cheRNA) class of gene regulatory non-coding RNAs was identified. The computational framework presented here provides a reliable approach to identifying cheRNAs from nuclear RNA-seq, and for studying cell-type specific gene regulation. We find that intergenic cheRNA, including transcripts mapped to regions with high levels of classically repressive H3K9me3-marks, may act as a transcriptional activator. In contrast, antisense cheRNA, which originates from the DNA strand complementary to the candidate target protein-coding gene may interact with diverse chromatin modulators to repress local transcription. Our new pipeline allows the identification of a more complete set of cheRNAs than other approaches. A future challenge will be to refine the functional mechanisms of cheRNAs by exploring their regulatory roles, which are involved in diverse molecular and cellular processes in humans and other organisms.

## Introduction

Both the nucleoplasm and the chromatin fraction of the nucleus are enriched in long noncoding RNA (**lncRNA**) [1]. Many nuclear lncRNAs affect coding gene expression, alter chromatin organization, and are important in diverse biological processes [2, 3]. Among these nuclear lncRNAs, chromatin-enriched RNAs (**cheRNAs**) possess gene-regulatory roles [4–7]. In our recent studies, we found individual cheRNAs that promote essential gene-enhancer contacts are dependent on a transcript factor [5, 7]. However, a robust analytic pipeline for the identification of cheRNA as a group of functional nuclear RNAs has not been developed.

Improvements in the bioinformatic analysis of nuclear RNA-seq are required because the distinctions between mRNA-seq and nuclear RNA-seq are substantial. The differences in library construction have significant consequences for the interpretation and analysis of the sequencing data [8]. For instance, sequencing of polyadenylated (**polyA+**) RNA may miss transcripts that are not polyadenylated and include many lncRNAs. Total-RNA sequencing detects a higher proportion of lncRNA, but is more expensive and less efficient in quantifying coding-gene expression [9].

In this study, we compare one published and three new analytic pipelines for nuclear RNA-seq data analysis. A newly developed pipeline, Tuxedo-ch, outperforms the other pipelines with respect to transcriptome completeness, accuracy of cheRNA identification, and enrichment of enhancer-hallmarks at cheRNA loci. Based on the GENCODE annotation (v25) [10], we verify that cheRNA transcripts define a new class of putative *cis*-regulatory RNAs, which may be a type of enhancer RNA (**eRNA**).

We explore the association of identified cheRNA with chromatin marks and unveil possible regulatory functions of intergenic cheRNAs transcribed from locations adjacent to condensed chromatin (marked by H3K9me3) and of cheRNA transcripts antisense to coding genes. Surprisingly, our data indicate that cheRNA-marked eRNAs are located in regions surrounded by high H3K9me3, which is primarily associated with transcript repression. Given that H3K9me3 is associated with the silencing of mobile elements [11, 12], our data agrees with the theory that enhancers are co-opted from ancient mobile elements [13]. Another subgroup of cheRNAs, as-cheRNA, which are defined as being located antisense to coding genes, show distinct negative correlations in transcription level with their corresponding sense mRNAs. Our approach affords a straightforward method to identifying and predicting novel regulatory lncRNA for future mechanistic evaluation.

## Results

### Nuclear RNA-seq requires rigorous computational strategies

**Fig 1:**
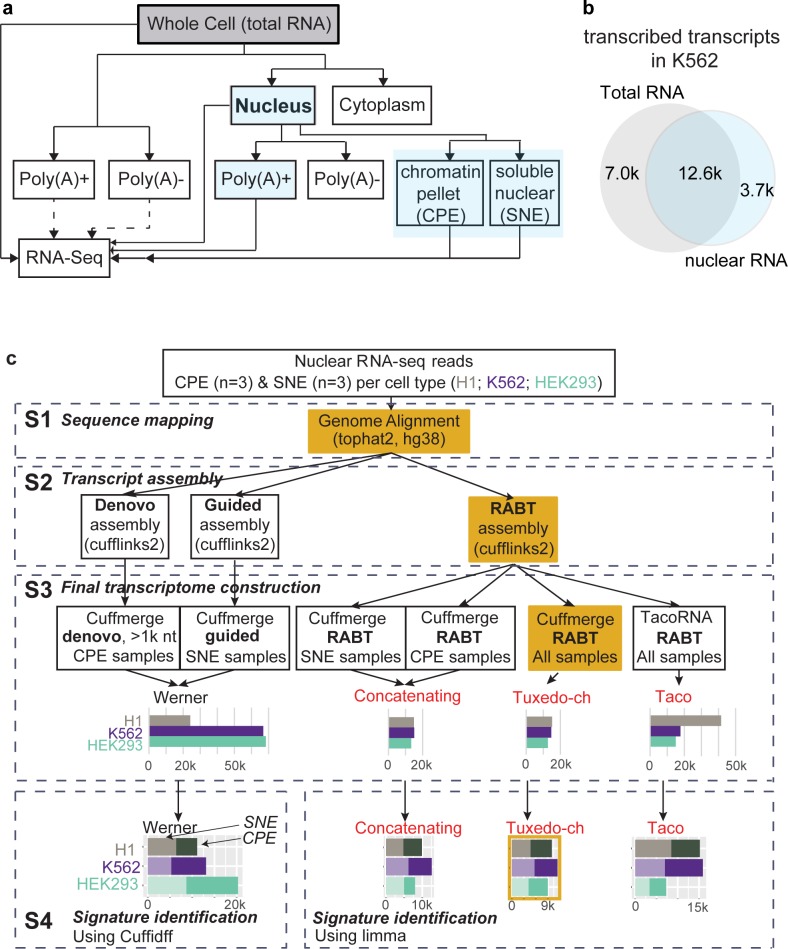
Nuclear RNA-seq sheds new insights into *cis*-regulatory elements. **a,** Diverse RNA-seq library strategies from parallel samples. Solid lines are the sequencing libraries (in category) analyzed in panel b, and dashed lines are other available libraries. Blue color indicates the nuclear RNA-seq strategies. **b**, Venn diagram compares the number of predicted transcripts in two pooled RNA-seq transcriptomes (K562 cells). One is the total RNA-seq transcripts that were expressed with ENCODE transcript quantification value>0 in both replicates, in at least one of four collected samples; and the other is the three types of nuclear RNA-seq transcripts (blue boxes in panel a, pooled from 10 samples, see **S1 Table**). The latter includes either all detectable transcripts (those with non-NA values in the downloaded data) in both its replicates, or expressed transcripts with CPM ≥ 1 in the Tuxedo-ch assembly. **c**, Workflow of the four nuclear RNA-Seq analytic pipelines with four major analytic steps: **S1,** Sequence mapping. **S2,** Transcript assembly. **RABT**: reference annotation-based transcript, which assembles both known and novel transcripts. **S3,** Final transcriptome construction. Bar plot represents the number of all expressed transcripts (Werner: without NA statistics in Cuffdiff; other three pipelines: CPM≥1); color indicates the assembly result in different cell line. **S4**, Signature identification. Stacked bar plot represents the number of RNA with different abundance in the CPE (darker color) or SNE samples (lighter color).

Nuclear RNA-seq library construction differs from other RNA-seq protocols (**Fig 1A**). For example, the numbers of detected transcripts differ when nuclear or total RNA is sequenced, with 30.0% (7.0 k out of 23.3 k) of the transcripts detected only by total RNA sequencing, and 15.9% only by nuclear (**Fig 1B**). This difference is unlikely to be simply due to sequencing depth—the median depth was 49M for four pooled total RNA samples and 33M for 22 nuclear RNA samples; the latter includes the 9 Chromatin Pellet Extract (**CPE**) and 9 Soluble Nuclear Extract (**SNE**) samples reanalyzed in this study (**S1 Table**). Markers of transcriptional regulation including RNA polymerase II (**Pol II**) sites, transcription factor binding sites, *cis*-regulatory RNA structures, histone deacetylase, and histone enhancer hallmarks are common in the DNA corresponding to the 3700 RNAs detectable only by nuclear RNA-seq (**S1 Fig, S2 Table**). This observation agrees with previous suggestions that nuclear-retained lncRNA may interact with chromatin regulatory proteins and recruit them to *cis*-regulatory elements in order to influence gene transcription [1, 3]. We conclude that sequencing distinct pools of RNA will detect different aspects of the transcriptome. Thus, analyzing only total RNA or mRNA can lead to missing portions of nuclear transcripts.

Conventional RNA-seq pipelines would not work for the identification of cheRNA that consists of both annotated and novel transcripts. After the isolation of RNA and generation of sequencing libraries, a typical RNA-seq analytic workflow involves sequencing hundreds of millions of reads, alignment of reads to a reference genome or transcriptome, and downstream statistical analysis of expression. In the original method, developed by Werner *et al*. [4, 5], which we refer to as Werner, cheRNAs were identified in four steps. This Werner pipeline has three important biases: 1) Werner overestimates the proportion of *de novo* transcripts originating from the CPE because it applies reference-guided *de novo* assembly (which can discover novel transcripts) to the CPE but not to the SNE fractions. 2) Werner removes transcripts shorter than 1000 bases from the analysis. LncRNA transcripts are typically shorter than (median length 592 bases) protein-coding transcripts (median 2.4k bases), and 26.8% of GENCODE-annotated lncRNAs (whose biotypes are not ‘protein-coding’) are longer than 200 but shorter than 1000 bases [14]. Removing transcripts shorter than 1,000 bases from the CPE assembly leads to significant under-detection of lncRNA. 3) In the differential expression analysis, Cuffdiff was used in Werner. However, Cuffdiff cannot do a two-group test on RNAs that have high expression levels. For example, the long noncoding RNA *XIST* was categorized as “HiDATA” and excluded from differential expression analysis by Cuffdiff. In addition, it has been shown that discarding genes that are not expressed at a biologically meaningful level in any condition (prefiltering) can increase the power for detecting differential gene expression [15], but Werner does not include a prefiltering step in the differential expression analysis.

There is a critical need for a rigorous and effective protocol to analyze nuclear RNA-seq, different from the classic schemes used for mRNA-Seq or total RNA-seq datasets. We have developed new pipelines considering two principles. First, there is no one-size-fits-all pipeline for data analysis; therefore, an empirical approach is recommended for noise removal. Second, biological conclusions need to be data-driven; therefore, both the CPE and SNE samples are analyzed using the same strategy. Specifically, the inclusion of relatively shorter (<1000 bases) lncRNAs allows for greater systematic discovery.

### Tuxedo-ch outperforms three existing and alternative analytic pipelines

#### Tuxedo-ch builds a complete transcriptome for active transcripts

We developed three new pipelines (referred to here as Tuxedo-ch, Concatenating, and Taco) to analyze these datasets; each with four major analytic steps (**Fig 1C**). Detailed discussion of the theoretical aspects of the four nuclear RNA-seq analytic pipelines is given in the **S2 File**. The Tuxedo-ch pipeline makes two key computational improvements: 1) Tuxedo-ch assembles the complete transcriptome in an unbiased way, covering both highly-expressed transcripts and lncRNA shorter than 1,000 bases. 2) Tuxedo-ch employs an empirical threshold to distinguish low but informative lncRNA transcription from noise. As a result, for the first time, we report a correlation between the expression of icheRNA and adjacent genes, facilitating the prioritization of potentially *cis*-acting cheRNA for further experimental evaluation. The strategies used in the Tuxedo-ch pipeline are not restricted to cheRNA identification and could be beneficial to nuclear RNA-seq data analyses testing broader biological hypotheses, such as to the relationship between enhancer marks (*e*.*g*., **S1 Fig**) and differentially expressed nuclear RNA.

**Fig 2:**
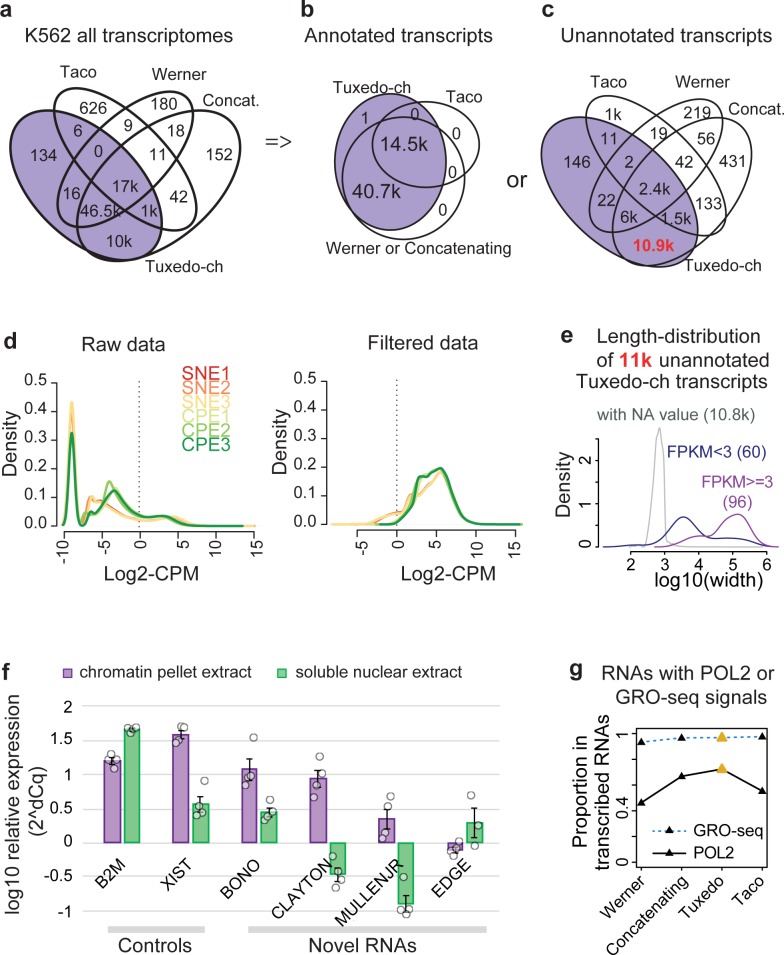
Tuxedo-ch assembles a complete high quality transcriptome. **a**, Coordinate-overlap for all transcripts in predicted RNA classes in the K562 cell. Numbers are counted by the R package ChIPpeakAnno with the “findOverlapsOfPeaks” function. RNAs with an overlap of 1 base or more are considered to be overlapped. If multiple transcripts overlap in several groups, the minimal number of transcripts in any group is shown. **b**, Coordinate-overlaps for annotated or **c,** unannotated RNA, being respectively constructed by 4 pipelines. **d**, Density plot showing the consequence of filtering the lowest-expressed values by the Tuxedo-ch pipeline. A nice bell-like shape of count distribution is observed after this filtering of noise. Line colors decode individual samples. **e**, Length distribution of unannotated RNAs only predicted by Tuxedo-ch, which were spited into three categories. Those shorter transcripts with NA FPKM values (grey population) were all excluded by the noise-removal step in panel d. **f**, RT-qPCR experiment validating the transcription of four novel noncoding RNAs using 4 CPE and 3 or 4 SNE biological replicates in K562 cells. Data are normalized to a unique, in-vitro transcribed spike-in RNA standard (ERCC standard #42). Bars represent the mean and standard error bars (n = 4), while circles show individual replicates. **g**, Proportion of expressed RNA (CPM ≥ 1) assembled in each pipeline that overlap (at least 1 base, same strand) by coordinate with any GRO-seq peak and POL II peak.

To evaluate the transcriptomes assembled by the four pipelines, we used the assembly results of K562 cell data as a reference. We first compared the completeness of the transcriptomes. Transcriptomes assembled by the Tuxedo-ch and Concatenating assemblies were very concordant. 99.8% of transcripts were the same. 84.4% of transcripts assembled by both Tuxedo-ch and Concatenating were also assembled by Werner. This number decreased to 27.0% for Taco (**Fig 2A**). To determine the reasons for assembly inconsistency, we compared the assembly results for annotated transcripts (**Fig 2B**) and novel transcripts (**Fig 2C**) separately. The annotated transcripts assembled by Tuxedo-ch, Concatenating and Werner pipelines were almost identical, and correspond to the set of annotated transcripts in GENCODE (v25). Taco only assembled 26.2% of annotated transcripts. This is because the Taco pipeline uses TACO instead of Cufflinks as the assembly tool. TACO only includes a small fraction of annotated transcripts that have significant expression, while Cufflinks keeps all annotated transcripts when building the transcriptome. Among the unannotated transcripts assembled by Tuxedo-ch and Concatenating, 96.8% were the same. Taco also assembled the smallest number of unannotated transcripts. In summary, Tuxedo-ch and Concatenating assembled more novel transcripts than Werner and Taco.

We next investigated the length of all assembled transcripts (**S2 Fig, S2 File**). The length distribution of transcripts assembled by Tuxedo-ch and Concatenating, but not by Werner, showed that the majority of such transcripts are shorter than 1000 bases (**S2A Fig**). Transcripts assembled by Werner are mostly longer than 1000 bases since this pipeline removed shorter transcripts from CPE samples. Transcripts assembled by Taco were much shorter compared to the other pipelines (**S2B Fig**). The TACO assembler employs an algorithm based on change-point detection via binary segmentation to predict transcript structure [16] but may overestimate the degree of alternative splicing and results in a large number of truncated transcripts. This is incorrect since only a small fraction of lncRNAs undergo splicing [17]. In summary, Tuxedo-ch and Concatenating construct relatively comprehensive and correctly structured transcriptomes for analysis.

Given that lowly expressed transcripts are likely to be experimental noise [18], we adopted Tuxedo-ch to identify lncRNAs with low expression. Unlike methods applied to coding gene profiles, in which one can define an expression cutoff for active promoters, we made an empirical decision: transcripts with counts per million (**CPM**) ≥ 1 in at least half of the samples were defined as ‘expressed’ for downstream analysis. This filter resulted in an approximately log-normal distribution of expression levels and about 14 k measured transcripts per sample (**Fig 2D**). Only 1.4% (156) of the 11k Tuxedo-specifically assembled novel transcripts passed this filter; the longer (>10k bases) transcripts often had higher expression values (**Fig 2E,** average Fragments Per Kilobase of transcript per Million mapped reads (**FPKM**) ≥5). The observation that almost all filtered novel transcripts are shorter than 1k bases is reminiscent of a previous work where transcripts shorter than 1k bases were discarded under the assumption that shorter transcripts are more likely to represent spurious transcription [5]. However, rather than using a hard cutoff of transcript length, we recommend a data-driven noise-removal strategy to select expressed transcripts more comprehensively and with less bias.

#### Tuxedo-ch identifies novel lncRNAs

We validated the transcription of these Tuxedo-ch discovered novel transcripts. Reliable detection by RT-qPCR supports that the RNAs targeted by specific primers are indeed transcribed. The RT-qPCR evaluation of four biological replicates from chromatin-pellet extract and soluble nuclear extract of K562 cells confirmed the transcription and nuclear localization patterns of four RNAs randomly selected from all 156 novel transcripts (**Fig 2F, S3 Table**). We refer to these four novel transcripts as BONO (Chr12:91,338,266–91,634,409), CLAYTON (Chr17:22,090,687–22,205,154), MULLENJR (ChrX:121,191,887–121,869,550), and EDGE (Chr6:6,683,958–6,686,966). One validated transcript, EDGE, is relatively short (3k bases) with a low expression level (average FPKM = 4.5). We conclude that Tuxedo-ch can construct a comprehensive transcriptome including those novel lncRNAs.

Additionally, we compared the transcriptional activity of the expressed transcripts assembled by the four pipelines using two independent measurements: Pol II ChIP-seq and global run-on sequencing (**GRO-seq**) (**S1 Table**). Expressed transcripts were defined as those having CPM ≥ 1. The expressed transcripts assembled by Tuxedo-ch showed the highest proportion of overlap with peaks representing both ongoing transcription by Pol II and peaks representing nascent transcription by GRO-seq (**Fig 2G**), demonstrating that expressed transcripts assembled by Tuxedo-ch are more concordant with the active transcription signals indicated by other methods. In summary, we introduce the computational pipeline, Tuxedo-ch, for analyzing nuclear RNA-seq data containing both high and low lncRNA expression.

#### Tuxedo-ch identifies cheRNAs while recapturing three known genomic features of active enhancers

**Fig 3:**
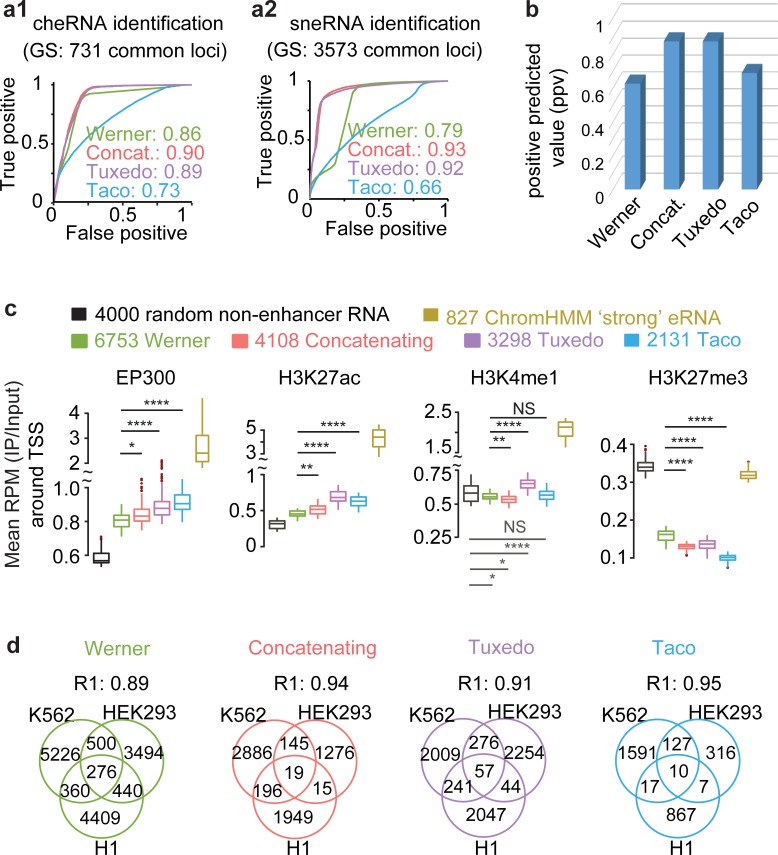
cheRNA prediction using the four pipelines in the K562 cell line. **a,** Receiver operating characteristic (ROC) curves of four pipelines identifying cheRNA (a1) and sneRNA (a2). The commonly-identified 731 cheRNA or 3573 sneRNA by all four pipelines are the proxy gold standard (GS) used here. **b**, Average positive predicted value (**PPV**) in identifying sixteen canonical cheRNA/sneRNA/intermediate RNA (RNA not differentially expressed between CPE and SNE) experimentally verified in previous studies for chromatin-enrichment or depletion, using the four pipelines, respectively. Further details about these 16 loci are given in **S4 Table**. **c**, Average ChIP-seq read density around TSS (±1kb centered at TSS) of the indicated RNA classes in K562. Boxes span the lower to upper quartile boundaries, the median is indicated with solid line in each box. ChromHMM-predicted eRNA is defined as intergenic RNA overlapped (at least 1 base, same strand) with any ChromHMM predicted “strong enhancer” region and ChromHMM-predicted non-enhancer RNA is defined as transcribed RNA that have no overlap with any ChromHMM-predicted “strong” or “weak” enhancers. **d,** Fraction of cell-type-specific intergenic cheRNAs. R1 is the ratio of cell type specific RNAs versus all RNAs identified in each pipeline. Higher R1 value indicating more cell-type specific identification. Venn diagrams show the overlap of icheRNA identified in K562, HEK293 and H1-hESC cell lines by Werner (green), Concatenating (red), Tuxedo-ch (purple) and Taco (blue). icheRNA identified by all the four pipelines except Werner (green) have high tissue-specificity (R1>0.9).

To evaluate the performance of the four pipelines in cheRNA identification, we used a set of transcripts identified by all methods as a proxy gold standard to perform Receiver Operating Characteristic (**ROC**)-evaluation. With the sets of 731 common cheRNA predictions and 3.6k Soluble Nuclear Extract RNA (**sneRNA**) predictions, Tuxedo-ch and Concatenating outperformed Werner and Taco in the identification of both cheRNA and sneRNA (**Fig 3A**). To further check the accuracy we examined sixteen loci of known cheRNA, sneRNA, or chromatin-independent RNA (transcripts not significantly differentially expressed between CPE and SNE samples) that were previously validated in specific cell types [4, 5]. Tuxedo-ch and Concatenating successfully confirmed the chromatin enrichment of all canonical cheRNAs and outperformed Werner and Taco with an overall positive predicted value (**PPV**) of 0.88 (**Fig 3B, S4 Table**). This PPV analysis, although possibly susceptible to threshold effects, makes up for the shortage of a ROC-analysis due to the lack of a true gold standard. Both analyses suggest that Tuxedo-ch and Concatenating are better than Werner and Taco.

Because intergenic cheRNA (**icheRNA**), which is defined as cheRNA without coordinate overlap with known coding genes, is similar to eRNA [4, 5], we examined the occupancy of enhancer marks (ChIP-seq signals of EP300, H3K27ac, H3K4me1) and a repressive mark (H3K27me3) around the TSS of the 2.0 k to 6.7 k icheRNA identified by each pipeline (**Fig 3C**). In this analysis, we used the broad Chromatin state segmentation by Hidden Markov Model (**ChromHMM**)-predicted eRNA (**Fig 3C**, yellow) and non-enhancer RNA (**Fig 3C,** black) as positive and negative controls [19]. ChromHMM-predicted eRNA was defined as intergenic RNA that overlaps (at least 1 base, same strand) with any ChromHMM predicted “strong enhancer” region and ChromHMM-predicted non-enhancer RNA was defined as transcribed RNA that had no overlap with any predicted “strong enhancer” or “weak enhancer” region. We found that the levels of enhancer marks (EP300, H3K27ac) were significantly higher around the TSS of the icheRNA identified by Tuxedo-ch (**Fig 3C**, purple) compared to the TSS of ChromHMM predicted non-enhancer RNA (**Fig 3C**, black), while the levels at icheRNA of repressive marks was significantly lower. At TSSs, we observed that cheRNA identified by all four pipelines has higher H3K27ac and EP300 signals than random non-enhancer RNA, but not H3K4me1 marks. These observations connect cheRNA TSSs to active transcription regulation; while the ENCODE H3K4me1 data may lack antibody specificity or be also associated with inactive enhancers [20, 21]. We also noticed that the levels of enhancer marks around TSS of ChromHMM-predicted eRNA, the benchmarking controls, were much higher than those around TSS of icheRNA, possibly because the enhancers predicted by ChromHMM are biased toward those having high occupancy of canonical enhancer marks. Additionally, all three new pipelines presented similar cell-type specificity compared to Werner, as evaluated by the proportion of tissue-specific icheRNA identified by each pipeline (represented by R1 score in **Fig 3D**). In summary, icheRNAs identified in nuclear RNA transcripts are associated with eRNAs with higher H3K27ac and EP300 occupancies. Among the four nuclear RNA-seq analytic pipelines, Tuxedo-ch achieves the strongest enrichment of enhancer hallmarks at icheRNA.

Overall, we conclude that Tuxedo-ch and Concatenating outperform the other two pipelines in identifying expected cheRNA, and that the Tuxedo-ch predicted icheRNA transcripts are more highly enriched in enhancer hallmarks compared to other methods. Therefore, we employ Tuxedo-ch to characterize different subclasses of cheRNA as described below.

### Intergenic cheRNAs (icheRNA) uniquely present eRNA features

#### icheRNA represents a subset of noncoding RNAs de novo

**Fig 4:**
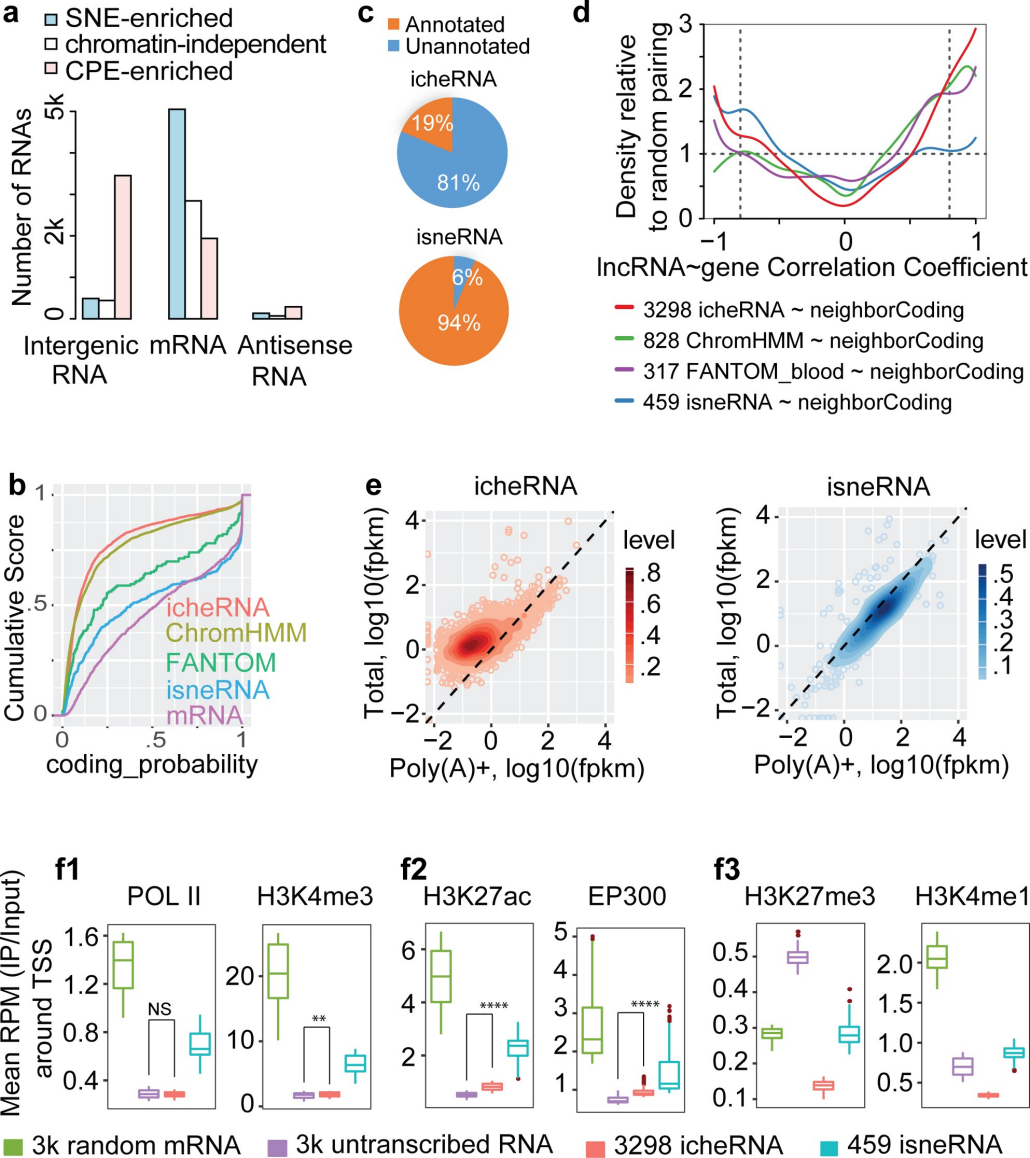
Known genomic features of the icheRNA in the K562 nucleus. **a**, Distribution of three classes of expressed RNA (CPM>1) in fractionated libraries. Three classes were defined based on their relative genomic locations to GENCODE (v25)-annotated coding genes (**S3 Fig**). Chromatin-independent RNAs refer to RNAs not differentially expressed in CPE and SNE samples. **b,** Coding potential of icheRNA (red), ChromHMM-predicted intergenic eRNAs (yellow), FANTOM5-predicted eRNAs (green, of which the majority overlap with coding genes), isneRNA (blue) and mRNAs (purple). Intergenic RNA overlapped (at least 1 base) with any ChromHMM/FANTOM5 identified enhancer region is assumed to be predicted ChromHMM/FANTOM5 predicted eRNAs. The online tool CPC2 is used. **c,** Percentage of GENCODE (v25) annotated and unannotated RNAs in icheRNA and isneRNA. **d,** Pairwise Correlation of expression between RNAs in four classes and their local genes. To pair an intergenic genomic feature with its neighboring gene, the adjacent upstream or downstream gene with the highest magnitude PCC is selected. The relative density at a certain PCC value is calculated by dividing the kernel density estimates of indicated RNA and neighboring-gene pairs by its own random control. For example, the random control of the 3,298 icheRNA-neighboring gene pairs is that of pairing these icheRNA with randomly selected coding genes (**S4 Fig**, dash line). Two vertical dashed lines mark significant cutoffs of PCC values at -0.8 or 0.8. **e,** Normalized expression values of fractionate RNA classes. Values are given in FPKM in Poly(A)+ nuclear RNA-Seq library (x-axis, GSE88339) versus nuclear total-RNA-Seq library (y-axis, GSE87982). **f**, Average ChIP-seq read density versus input centered at promoters (±1kb centered at TSS) of RNAs, *p*-values calculated by two-sided Wilcoxon rank sum test, NS p>0.05, * p<0.01, ** p<1e-10, **** p<2.2e-16. (Note that in each panel, boxes without overlaps are significantly different without showing **** for simplicity.) 3k random mRNAs were selected from 9.8k transcribed mRNAs and 3k randomly selected silent RNAs from 66.9k annotated but K562-untranscribed RNAs.

Werner *et al* proposed that icheRNA is a distinct class of unannotated and gene-activating lncRNA. To further examine this hypothesis, we categorized the 14k expressed nuclear RNAs detected by Tuxedo-ch into three groups: intergenic RNA, lncRNA transcribed antisense to a coding genes (labeled as “antisense RNA” in **Fig 4A**), and those that overlap with mRNAs in the sense orientation (labeled as “mRNA” in **Fig 4A**). **S3 Fig** shows the workflow used to categorize the three RNA groups). A large fraction (66%) of the 5,680 identified cheRNAs were transcribed from noncoding regions (**Fig 4A,** pink bar). In contrast, approximately 90% of the identified 5,672 sneRNAs were mRNAs (**Fig 4A**, blue bar). Additionally, K562-cell data show that icheRNA exhibited lower coding potential (cumulative Coding Potential Calculator 2 (**CPC2**) score [22]) than coding genes, intergenic sneRNA (**isneRNA**), and intergenic RNA transcribed from ChromHMM-predicted enhancer regions or FANTOM5-predicted eRNAs that mostly overlap with genes by cap analysis gene expression (**CAGE**) [23] (**Fig 4B**). The coding potential of icheRNA is therefore more similar to that of ChromHMM-predicted intergenic eRNA, while that of isneRNA is more similar to that of mRNA.

81% (2.7 k) of the identified 3.3 k icheRNAs were previously unannotated transcripts, in contrast to only 6% (27) of the 459 isneRNAs, (**Fig 4C**). Additionally, over half (69% of 445) of the antisense cheRNAs were unannotated, in contrast to only 2% of 163 antisense sneRNAs. This biased annotation of noncoding RNA suggests that previously detected noncoding RNAs primarily correspond to chromatin-depleted noncoding RNA (i.e., noncoding sneRNA), and that identifying chromatin-enriched RNAs from nuclear extracts can give a more holistic picture of the overall noncoding RNA population.

#### icheRNA positively correlate with adjacent genes in expression

RT-PCR experiments have shown that several expressed eRNAs are intergenic icheRNA [7]. Werner *et al*. also showed that protein-coding genes proximal to icheRNA have higher expression levels than those near other expressed lncRNA, suggesting that icheRNA could predict local *cis*-gene transcription [4, 5]. However, it is not clear from previous work whether higher icheRNA expression is correlated with expression of proximal protein-coding genes. To quantitatively confirm the *cis*-regulatory potential of icheRNA, we calculated the Pearson correlation coefficient (**PCC**) between the expression of icheRNA and neighboring protein-coding genes and compared it to the simulated PCC between the expression levels of icheRNA and randomly selected protein-coding genes. Similarly, we calculated these two types of PCCs for isneRNA, ChromHMM predicted eRNA, and FANTOM5 predicted eRNA, respectively.

We found that the expression of icheRNAs is more positively correlated with the expression of neighboring genes than with randomly selected genes (**Figs 4D** and **S4,** red line shows relative density > 1 when correlation coefficient > 0.5). The same calculation for FANTOM-predicted eRNAs or ChromHMM predicted intergenic eRNAs, which are believed to have *cis*-regulatory enhancer effects, and adjacent genes in the same cell types, showed similar but weaker positive correlations. In contrast, pairs of isneRNA and neighboring genes (blue line) showed a negative correlation (**Fig 4D**, blue line shows relative density > 1 when correlation coefficient < -0.5). Specifically, with a significance cutoff of correlation coefficient = 0.8, 23% of the identified icheRNA transcripts, in contrast to only 11% of the isneRNA were positively correlated with proximal genes. This observation, for the first time, gives quantitative evidence for a potential *cis*-regulatory effect of icheRNA on adjacent genes. It also suggests that icheRNA identification as a new approach to predict *cis*-activating lncRNA, comparable to the conventional approaches using the ChromHMM or FANTOM5 databases.

Transcriptional correlation analysis also displayed a high relative density at correlation coefficient < -0.5 for pairs of icheRNA and neighboring coding genes (**Figs 4D and S4)**, indicating that not all icheRNAs are positively correlated with proximal protein-coding gene expression. Indeed, *XIST* is a well-known icheRNA that has a repressive regulatory role, and it might be one of the icheRNAs that are negatively correlated with proximal protein-coding genes.

#### icheRNA is depleted in polyadenylated RNA

Most eRNAs have been reported to be unspliced and non-polyadenylated [24–27]. To test if icheRNA are similar in this regard, we compared the relative expression (measured as Fragments Per Kilobase of transcript per Million mapped reads, **FPKM**) of intergenic cheRNAs in a nuclear Poly(A)+ RNA-seq library and nuclear total RNA-seq libraries using published datasets for K562 cells (**S1 Table**). We observed (**Fig 4E**) higher expression of icheRNA in the nuclear total-RNA library and lower relative abundance in the nuclear Poly(A)+ RNA-seq library, supporting the conclusion that icheRNA are relatively weakly polyadenylated. A similar, but weaker, preference for the total-RNA-seq library was also observed for antisense cheRNAs (**S5 Fig**). In contrast, all protein-coding mRNAs had equivalent expression levels in two libraries, which is consistent with the role of polyadenylation in producing mRNA in the eukaryotic cell nucleus [28]. Chromatin depleted non-coding RNAs (isneRNA and antisense sneRNAs) also had similar expression levels in the two RNA-seq libraries (**S5 Fig**). The patterns of polyadenylation indicate that icheRNA and isneRNA are differentially polyadenylated. With respect to polyadenylation, icheRNA appears to be more similar to eRNA than is sneRNA, since the majority of icheRNA are depleted in the Poly(A)+ RNA-seq library.

#### icheRNA and isneRNA loci have different chromatin characteristics

Histone 3 lysine 4 monomethylation (H3K4me1) and histone 3 lysine 27 acetylation (H3K27ac) have been identified as key histone modification features that mark enhancers. H3K4me1 is present at both poised and active enhancers [29], while H3K27ac uniquely marks active enhancers [30]. Werner *et al*. previously observed peaks of H3K27ac near the transcriptional start sites (**TSS**) of icheRNA, however, unlike prototypical eRNA, these regions did not show abundant H3K4me1 modification [4]. To further investigate whether icheRNA have a distinct chromatin signature, we profiled the relative reads per million (**RPM**) of RNA polymerase II (POLII), H3K27ac, H3K4me3, H3K4me1, and H3K27me3 marks on the flanking 1 kb sequences around TSSs of icheRNA, isneRNA, mRNA and unexpressed mRNA (RNAs annotated in GENCODE(v25) but not transcribed in K562) (**Fig 4F**). IcheRNA showed low levels of marks associated with active transcription (POLII and H3K4me3), similar to the levels of unexpressed mRNA, and lower than those of isneRNA and mRNA (**Fig 4F1**, red and purple box). In contrast to unexpressed mRNA, icheRNA TSS flanking regions showed low levels of repressive (H3K27me3) and poised enhancer (H3K4me1) marks (**Fig 4F3,** red and purple box), and were enriched in active enhancer (H3K27ac and EP300) marks (**Fig 4F2,** red and purple box). In addition to being enriched at enhancer regions, H3K27ac and EP300 marks were also pervasively found near TSS of actively transcribed regions. IcheRNA TSS thus have a chromatin profile that is distinct from those of mRNA, isneRNA, and unexpressed mRNA, suggesting that significantly different modes of regulation may be controlling icheRNA expression.

In summary, icheRNA and isneRNA differ in many respects. In addition to the enrichment of specific epigenetic marks near the TSS, icheRNA has lower coding probability, appear to lack polyadenylation, and icheRNA expression is more positively correlated with that of neighboring coding genes. Overall icheRNA is more similar to eRNA, while isneRNA is more similar to mRNA. The similarity of icheRNA to eRNA, as defined by ChromHMM and FANTOM5 predictions, suggests that icheRNA identification may provide a useful independent approach to predicting eRNA.

### *Cis*-regulatory potential of both icheRNA coincides with histone mark H3K9me3 and the cheRNA antisense to a coding gene

#### icheRNA with H3K9me3 signal across transcript bodies opts to be associated with active *cis*-regulation

**Fig 5:**
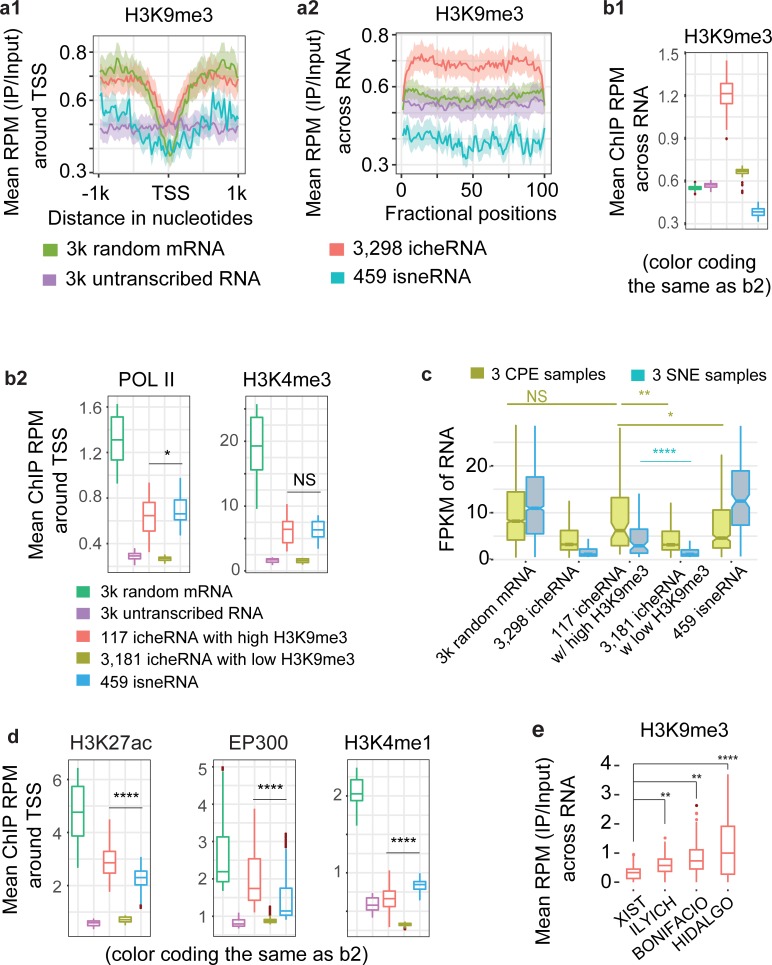
icheRNA with H3K9me3 signal concurs chromatin modification patterns of active enhancers. **a,** Average H3K9me3 ChIP-seq read density versus input in K562 cells (**a1**) at promoters (±1kb centered at TSS) of, or (**a2**) across transcript bodies. **b,** Average ChIP-seq read density versus input in K562 cells of (**b1**) H3K9me3 profiles across regions transcribing, or (**b2**) POL II and H3K4me3 profiles at promoters (±1kb centered at TSS) of five classes of RNAs (decoded in colors). **c,** Normalized expression values in FPKM in chromatin pallet extract (CPE, red boxes) and soluble nuclear extract (SNE, blue) of K562 cells for randomly selected mRNA, icheRNA, icheRNA with high H3K9me3, icheRNA with low H3K9me3 and isneRNA. **d,** Average ChIP-seq read density in K562 cells of active enhancer marks (H3K27ac and EP300) and poised enhancer mark (H3K4me1) profiles at promoters (±1kb centered at TSS) of five classes of RNAs. **e,** Average H3K9me3 ChIP-seq read density versus input in K562 cells across regions transcribing four canonical cheRNAs. The four cheRNAs were ordered according to their known transcriptomic regulatory functions, from the repressor (*XIST*) on the left to other three *cis*-activators on the right. P-values calculated by two-sided Wilcoxon rank sum test, NS p>0.05, * p<0.01, ** p<1e-10, **** p<2.2e-16.

Histone 3 lysine 9 trimethylation (H3K9me3) is associated with constitutive heterochromatin and has been shown to mark transcriptionally repressed regions that are mutually exclusive with H3K27me3 marked repressive regions [31–34]. We explored H3K9me3 occupancy at icheRNA loci. We conducted a meta-gene analysis, in which the average of the ChIP-seq signal within 2k-base window centered at the TSS within grouped transcripts (**Fig 5A1**) was calculated. Interestingly, we found that the levels of H3K9me3 near (100–1000 bases away from) actively transcribed icheRNA and mRNA TSS (**Fig 5A1**, red line and green line) were much higher than that near transcriptionally silenced TSS (DNA regions near to unexpressed mRNA) (**Fig 5A1**, purple line). In addition, H3K9me3 profiles at actively transcribed regions were quite different from those at transcriptionally silent regions. Specifically, H3K9me3 was high on either side of the TSS of transcribed RNA (icheRNA, isneRNA, and mRNA), but the signal drops precipitously at the TSS (**Fig 5A1**, red, blue and green lines). In contrast, H3K9me3 was flat around the TSS of unexpressed mRNA (**Fig 5A1**, purple line). It has been suggested, for coding transcripts, that H3K9me3 at the promoter is repressive, whereas H3K9me3 across the mRNA gene body is activating [32], consistent with the pattern we observed. These observations suggest that H3K9me3 modification at the TSS is associated with transcriptional silencing, whereas H3K9me3 modification within the gene body or peripheral to the TSS can be associated with active transcription.

We also noted that H3K9me3 levels within the DNA region of icheRNA were substantially higher than levels at other transcribed RNA (*e*.*g*., mRNA and isneRNA) (**Fig 5A2**). Comparing the average ChIP RPM across RNAs (**Fig 5A2**) to that around TSSs (**Fig 5A1**), the H3K9me3 values from icheRNA (red) and random mRNA (green) were substantially different, indicating a coincidence of H3K9me3 appearance alongside icheRNA transcripts. To further investigate the effect of H3K9me3 on icheRNA transcription, we separated DNA regions of icheRNA into high H3K9me3 (at least one peak of H3K9me3 mark near the transcribed icheRNA) and low H3K9me3 (no H3K9me3 mark near the transcribed icheRNA) groups. As expected, DNA regions in the “icheRNA with high H3K9me3” had significantly higher levels of H3K9me3 modification than those in the “icheRNA with low H3K9me3” group (**Fig 5B1**). The statistical significance of high H3K9me3 ChIP signal at regions peripheral to the TSS of the ‘high H3K9me3” group was true in two other groups of cheRNA, those overlapping with coding-genes on the same strand (P<2e-16, **S6A Fig**) or on the opposite strand (P<2e-16, **S6D Fig**). Interestingly, chromatin signatures associated with active transcription (POL2, H3K4me3) (**Fig 5B2**) were strikingly elevated in the “icheRNA with high H3K9me3” group compared to the “icheRNA with low H3K9me3” group around the TSS. This pattern was reproducible in the two other groups of cheRNA (**S6B and S6E Fig**).

We next checked the expression levels of transcripts in the chromatin pellet fraction and soluble nuclear fraction of which we had identified the cheRNAs and sneRNAs respectively (**Fig 5C**). The levels of icheRNA in the soluble nuclear fraction (the three blue boxes of icheRNAs in the middle), although significantly lower than that in the chromatin pellet fraction (each corresponding yellow box on the left), were also evaluated in the “icheRNA with high H3K9me3” group compared to the “icheRNA with low H3K9me3” group (P<0.01). The normalized expression level of the icheRNA with high H3K9me3 was similar to that of random RNA, suggesting an overall higher nuclear RNA transcription associated with local H3K9me3 occupancy. These observations reinforce the finding that regions with abundant H3K9me3 modification surrounding, but not at the TSS, can be actively transcribed. We conclude that icheRNA are more actively transcribed within regions with high surrounding H3K9me3 levels than those outside H3K9me3-marked regions.

Our previous analysis showed that icheRNA possesses features similar to eRNA. However, the TSS of icheRNA showed only moderately higher levels of enhancer marks compared to negative controls (annotated mRNAs unexpressed in the K562 cells) and lower levels than TSS of isneRNA. We measured the levels of enhancer marks (H3K27ac, EP300 and H3K4me1) around TSS of “icheRNA with high H3K9me3” (**Fig 5D**, red box). We found that levels of active enhancer marks (H3K27ac and EP300) around TSS of “icheRNA with high H3K9me3” were significantly higher than at the TSS of “icheRNA with low H3K9me3” (**Fig 5D**, yellow box) and TSS of isneRNA (**Fig 5D**, blue box). Moreover, we recaptured the higher levels of active enhancer marks around TSS of “coding-gene overlapped cheRNA with high H3K9me3” (**S6C Fig**), as well of “cheRNA antisense to coding-gene with high H3K9me3” (**S6F Fig**). We measured the H3K9me3 levels across canonical icheRNA transcribed regions and found that three previously identified icheRNAs (*HIDALGO*, *ILYICH*, *BONIFACIO*) with validated positive *cis*-regulatory activation function showed relatively higher H3K9me3 levels sounding their TSSs than the only icheRNA with a known repressive role (*XIST*) (**Fig 5E**). These examples reinforce the hypothesis that DNA regions transcribing icheRNA with high levels of surrounding H3K9me3 modification may act as enhancers.

#### Antisense cheRNA (as-cheRNA) co-occurs with local mRNA silencing (Fig 6)

**Fig 6:**
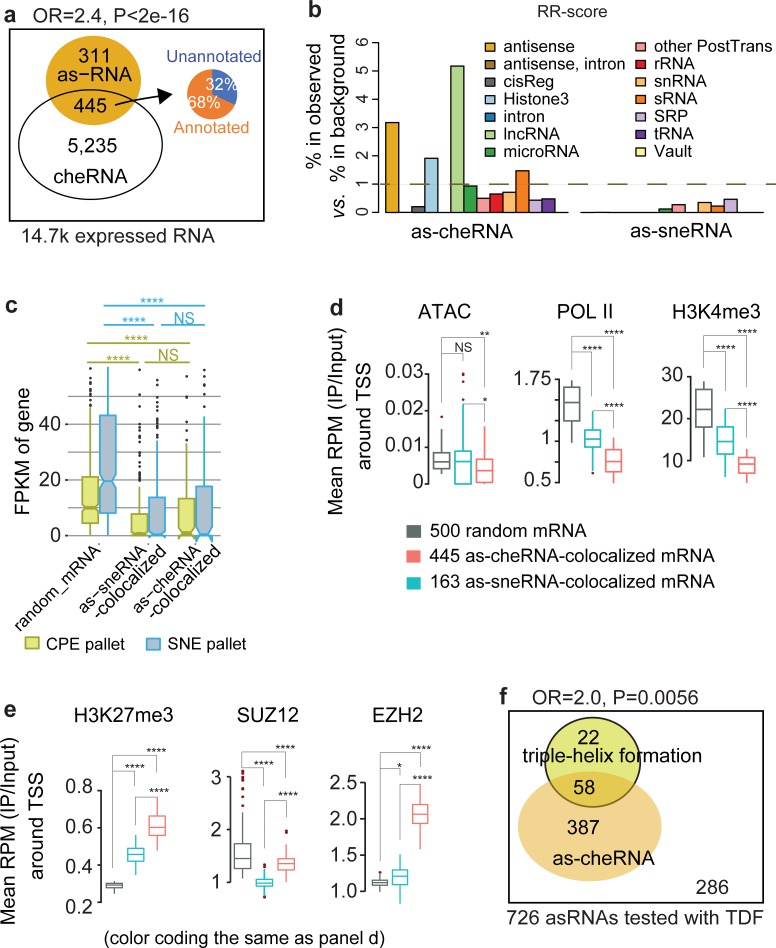
Antisense cheRNAs indicate local mRNA silencing. **a**, Venn diagram showing the enrichment between cheRNA and asRNA among 14.7k expressed transcripts in the K562 cells. Fisher’s exact test estimated the strength of enrichment (Odds ratio larger than 2 and p-value less than 0.05). Venn diagram showing Percentage of GENCODE (v25) annotated (orange) and unannotated (blue) RNA in as-cheRNA. **b**, Enrichment of 14 Rfam ncRNA secondary structure families among as-cheRNA (left sub-panel) and sneRNAs (right sub-panel). An RR-score larger than 1 indicates that as-cheRNA/as-sneRNA is overrepresented in the selected Rfam family. **c**, Normalized expression values in FPKM in chromatin pallet extract (CPE, yellow) and soluble nuclear extract (SNE, blue) of K562 cells for three RNA classes. **d**, Average ATAC-Seq read density and ChIP-seq read density of histone marks representing active transcription (POLII and H3K4me3) versus input in K562 cells at promoters (±1kb centered at TSS) of three RNA classes. **e**, Average ChIP-seq read density of repressive histone mark (H3K27me3) and two PRC2 subunits (SUZ12 and EZH2) versus input in K562 cells at promoters (±1kb centered at TSS) of three RNA classes. P-values are calculated using a two-sided Wilcoxon rank sum test, NS p>0.05, * p<0.01, ** p<1e-10, **** p<2.2e-16. **f**, Venn diagram showing the enrichment between cheRNA and triple-helix formation, among the 726 out of 756 asRNAs that had successful TDF-promoter tests. Fisher’s exact test was used to assess the enrichment.

Antisense RNA (**asRNA**) complementary to protein-coding transcript(s) has been shown to interfere with transcription of mRNA on the opposite strand [35]. Consistent with this, asRNA accumulated preferentially in the nucleus associating with chromatin: we observed that almost (59%) of the identified 756 asRNAs in K562 cell nucleus were chromatin-enriched and only 22% were chromatin-depleted (**Fig 4A**), indicating a significant enrichment of asRNA in the chromatin pellet (**Fig 6A**). Moreover, we noticed that about one third of the chromatin-enriched asRNAs (antisense cheRNA, **as-cheRNA**) were unannotated while 98% of chromatin-depleted asRNAs (antisense sneRNA, **as-sneRNA**) were annotated, suggesting that many as-cheRNA are completely novel.

Regulatory mechanisms involving asRNA range from simple transcriptional interference by competing for RNA Pol II [36] to regulation of epigenomic modifications [37, 38]. A current hypothesis suggests that asRNA acts in gene regulation at the chromatin level by recruiting epigenetic regulators, *e*.*g*., polycomb repressive complex 2 (**PRC2**), to its corresponding sense mRNA to induce histone methylation and gene repression [39, 40]. Inspired by this hypothesis, we first investigated one potential structure-based mechanism for both as-cheRNA and as-sneRNA. Functional RNA molecules often exhibit secondary structures that are better conserved than their primary sequences [41]. Therefore, we interrogated the multiple-sequence alignment-based families of RNA secondary structural motifs (**S7A Fig**), using the Rfam database [41]. Among fourteen major RNA structural groups in the Rfam database (v13, hg38), three structural groups (Histone 3, lncRNA, and antisense) were significantly overrepresented in as-cheRNA (**Fig 6B**, two or more folds). In particular, the overrepresentation of the antisense structure group among as-cheRNA suggests that the function of as-cheRNA, rather than that of as-sneRNA, is likely to be structure-based.

We then measured the transcription level in CPE and SNE of mRNA that antisense overlaps with as-cheRNA and as-sneRNA. We found that the transcription of both as-cheRNA-colocalized mRNA and as-sneRNA-colocalized mRNA were relatively low compared to that of random mRNA (**Fig 6C**), suggesting a negative correlation between the transcription of sense and antisense RNA. Even though both as-cheRNA-colocalized mRNA and as-sneRNA-colocalized mRNA were shown to be repressed at similar levels, the chromatin features and histone patterns around the TSS of the two mRNA groups were significantly different. The TSS of as-cheRNA-colocalized mRNA (**Fig 6D-e**, red box) were significantly less open (measured by ENCODE ATAC-seq signal), had fewer active transcription marks (POL2, H3K4me3), but had more repressive marks (H3K27me3), and showed higher PRC2 complex binding (EZH2 but not SUZ12) compared with random mRNA (**Fig 6E-e**, black box). This pattern was not observed in as-sneRNA-colocalized mRNAs (**Fig 6D-e**, blue box). Altogether, this raises the possibility that as-cheRNA and as-sneRNA may *cis*-repress local gene transcription through different mechanisms.

We further propose a molecular mechanism for the *cis*-repressive role of as-cheRNA, based on formation of RNA:DNA:DNA triple helixes that associate lncRNA and repressive chromatin (**S2 File**) [42]. Among the 756 asRNAs expressed in the K562 cells, the Triplex Domain Finder (**TDF**) analysis [43] predicted 80 possible triple-helix formations at gene promoters (TDF P<0.05). Primarily due to their extremely long transcript length, 30 asRNAs that were not run through the ‘TDF-promoter test’ were excluded from this analysis regardless of their chromatin-enrichment (**S2 File**, **S8 Fig**). Interestingly, 58 as-cheRNAs had predicted triple-helix formations, presenting a co-enrichment of as-cheRNA and predicted triple-helix formation in asRNAs expressed in K562 cells (**Fig 6F**, odds ratio = 2.0, P = 0.0056). This enrichment suggests that as-cheRNA represses transcription of adjacent mRNAs on the opposite strand, possibly by triple-helix formation.

## Discussion

Detailed analysis of nuclear RNA-seq from lncRNA that are shorter than 1,000 bases or transcribed at a low level sheds new light on *cis*-regulatory elements. Operationally, cheRNA are defined by their statistically significant enrichment in the chromatin pellet fraction after biochemical fractionation of nuclei. With our improved computational strategy, we have examined the molecular characteristics of cheRNAs in greater detail than has heretofore been possible. We find that, first, cheRNAs are more likely to be transcribed from noncoding regions, while sneRNAs are mostly transcribed from protein-coding regions. Second, icheRNA have a lower transcription level and are largely unannotated, in contrast with isneRNA which are more highly transcribed and more frequently annotated. Traditional transcriptome profiling of non-coding RNA, using techniques such as total RNA-seq, yields the broadest survey of transcripts, but has limited ability to detect low expression transcripts such as icheRNA. Thus, previous analyses of noncoding RNA primarily focused on noncoding RNA with relatively high transcription levels (*e*.*g*., isneRNA and as-sneRNA). In contrast, isolating and sequencing chromatin-enriched RNAs in a nuclear extract more sensitively identifies low expression noncoding RNAs that previously have been ignored by conventional sequencing and analysis methods. Third, we have shown that icheRNA, in contrast to isneRNA, is mostly non-coding, non-polyadenylated, and positively correlated with the expression of neighboring coding genes (**Fig 5A–5E**). Notwithstanding the similarity of these features to those of eRNA, icheRNA has several unique molecular characteristics that distinguish it. For example, icheRNA is generally longer than eRNA (median length of icheRNA is ~4,400 bases; eRNA is ~350 bases, [44]) and icheRNA shows only modest coincidence with enhancer marks (H3K27ac, H3K4me1 and EP300) that are used to canonically define eRNA (**Fig 4F**). Moreover, some icheRNAs (*e*.*g*., *XIST*) are known to be repressive regulators rather than activators. Combining all this evidence, we conclude that icheRNA more comprehensively defines chromatin-localized regulatory lncRNAs than *cis*-activating eRNA.

IcheRNA transcripts mapped to regions with high levels of H3K9me3-marks in the transcript body, but not at the TSS, are more actively transcribed and are more highly associated with elevated levels of enhancer marks than icheRNA transcribed from regions with low levels of H3K9me3. This observation indicates that paradoxically, the repressive H3K9me3 mark may be higher surrounding potential enhancer regions. The widespread presence of H3K9me3 at enhancer flanking regions has been previously observed [45]. Regulation of H3K9me3 levels specifically at the enhancers of Mdc and Il12b affected Mdc and Il12b transcription in dendritic cells and macrophages [45], suggesting that H3K9me3 plays an important role in regulating enhancer activity. If it can be verified that high flanking H3K9me3 is a common feature of enhancers, icheRNA coincident with H3K9me3 mark may prove a highly effective predictor for chromatin-based regulatory icheRNAs and may be a powerful approach to predicting novel enhancer regions.

Both icheRNA and transcribed mRNA show a substantial drop in the H3K9me3 signal at the TSS, although the degree to which the H3K9me3 signal is decreased at the TSS is much greater for mRNA than icheRNA (**Fig 5A1**). In metazoan cell nuclei, hundreds of large chromatin domains, termed Lamina-Associated Domains (**LADs**), are enriched in histones H3K9me2 and H3K9me3 modifications that are typical of heterochromatin [46]. A study on a 1 Mb LAD encompassing the human HBB loci showed that knockdown of H3K9me3 by depletion of the two H3K9me3 methyltransferases Suv39H1 and Suv39H2 caused detachment of the LADs and nuclear lamina [47]. Considering that H3K9me3 is a chromatin mark associated with closed/repressed chromatin, lncRNA transcription from H3K9me3-enriched LADs is expected to be repressed. However, the unexpected association between icheRNA and high levels of H3K9me3 chromatin marks suggests that icheRNAs may be embedded in, and actively transcribed from, condensed LADs. Indeed, we find that 48% of icheRNAs are transcribed from LADs (greater than chance expectation, empirical p < 2.2e-16). In contrast, only 12% of other RNAs are transcribed from LADs. Moreover, agreeing with the previous hypothesis by Werner *et al*. that transposable element may provide an evolutionary origin to chromatin-enriched RNAs [5], we noticed that 82% of icheRNAs and 96% of icheRNAs with H3K9me3 chromatin marks in K562 overlap with class 1 transposable elements (**TEs**). Together, these observations suggest that icheRNA may represent a group of RNAs transcribed from condensed chromatin domains derived from mobile elements [13].

Antisense RNA (asRNA) has been shown to have a *cis*-regulatory function. Consistent with previous knowledge, our analysis confirms that asRNA is more abundant in the nuclear chromatin pellet than in the soluble nuclear fraction. Similar to isneRNA and icheRNA, almost all as-sneRNAs are annotated, while a large fraction of as-cheRNAs lack annotation. This further suggests that sequencing RNAs abundant in the nuclear chromatin pellet can identify novel noncoding RNAs. Despite the fact that both as-cheRNA and as-sneRNA show negative correlations in transcription level with their corresponding sense mRNA, the chromatin pattern around the TSS of as-cheRNA-colocalized mRNA and as-sneRNA-colocalized mRNA are quite different. Regions around the TSS of as-cheRNA-colocalized mRNA are less open and lack active transcription marks, but have high a level of H3K27me3 and PRC2 binding, and high levels of predicted RNA:DNA:DNA triplexes. These genomic features suggest that as-cheRNA may regulate sense mRNA transcription by acting as a guide RNA for regulatory complexes that modify the target chromatin. Even though this investigation of as-cheRNA is preliminary, it provides some testable hypotheses for as-cheRNA function.

Nuclear fractionation coupled with RNA-seq provides a powerful way to profile the nuclear transcriptional landscape, especially the noncoding transcriptome. The computational pipeline presented here provides researchers with a reliable approach to identifying cheRNA, and for studying cell-type specific gene regulators. Although the cheRNA is unlikely to be monolithic in function, icheRNA, including icheRNA with high H3K9me3, may act as transcriptional *cis*-activators, of which eRNAs are a sub-group. In contrast, as-cheRNA may interact with diverse chromatin modulators to *cis*-repress transcription. With the Tuxedo-ch pipeline, the future challenge will be to refine the functional mechanisms of these noncoding RNA classes by exploring their regulatory roles, which are involved in diverse molecular and cellular processes in human and other organisms.

## Methods

**S1 and S2 Tables** list all publically available datasets analyzed in this study.

We compared three pipelines with the original cheRNA-identification pipeline [4]. Each pipeline includes four analytic steps: sequence mapping, transcript assembly, transcriptome construction, and signature identification (**Fig 1C**). Computational strategies in the latter three steps varied in four different pipelines (**S2 File**). Source file for the Tuxedo-ch pipeline is provided in the **S1 File**.

### Relative density of correlation between intergenic RNAs and neighbor coding genes (Fig 4D)

We calculated the pairwise Pearson correlation coefficient (**PCC**) between the intergenic RNA and protein-coding gene. We tested five types of intergenic RNA-gene groups: the icheRNA with random protein-coding gene pairs; the icheRNA with neighbor protein-coding gene pairs (icheRNA:neighborCoding); the broad Chromatin state segmentation by Hidden Markov Model (**ChromHMM**)-predicted eRNAs with neighbor protein-coding genes pairs (ChromHMM-neighborCoding), FANTOM5-predicted eRNAs with neighbor protein-coding genes pairs (FANTOM-neighborCoding) and the isneRNA with neighbor protein-coding gene pairs (isneRNA:neighborCoding).

The PCC of each intergenic RNA-gene pair was calculated based on their expression levels across all CPE and SNE samples of three cell types (K563, HEK293, H1-hESC). To pair an intergenic RNA with its neighbor protein-coding gene out of its nearest upstream and downstream genes on the same strand, the one with the highest absolute PCC value is selected. A significant cutoff of PCC values was set at -0.8 or 0.8, respectively for the negative or positive correlation. Kernel density is estimated for each intergenic RNA-gene pair group. Relative density for each intergenic RNA and neighboring protein-coding gene pairs group (*e*.*g*. icheRNA:neighborCoding) is calculated in the way of dividing the kernel density estimates of indicated intergenic RNA and neighboring protein-coding gene pairs group (*e*.*g*. icheRNA:neighborCoding) by the kernel density of icheRNA and randomly selected coding gene pairs group.

### RNA structural analysis (Fig 6C)

RNA structural analysis based on the Rfam annotations (v13, hg38) was conducted for each Rfam-family, by dividing its frequency within an ncRNA set (icheRNA or isneRNA) versus the frequency in all assembled RNAs. Each Rfam family is represented by a multiple-sequence alignment, a consensus secondary structure, and a covariance model [41]; and we grouped one or more annotating families into a super-family according to their function as well proportions in the above noncoding transcriptome (**S7A Fig)**. The homologous ncRNA sequences in each super-family were generally less than 400 bases (2.6 on the log10-scale, **S7B Fig**), much shorter than the ncRNAs in the assembled transcriptome. Therefore, we could annotate each asRNA as belonging to an Rfam family if it fully covered an Rfam family motif.

We then calculated, for each Rfam super-family (*r*), a) the fraction of the asRNA set (*t*) annotated to this Rfam family (observed fraction); b) the fraction of all assembled RNA annotated to this Rfam family (background fraction); and c) the ratio of the observed and background fractions (RR-score, Formula 1).

$$RR(t,r)=\frac{\left|t\;overlap\;r\right|}{\left|t\right|}/\frac{\left|t\;overlap\;T\right|}{\left|T\right|},$$ Formula 1

where *T* = {*t*} is the collection of all noncoding transcripts in the transcriptome, and |.| is the number of transcripts meeting a condition.

Therefore, an RR-score above 1 indicates an asRNA set (*t*)-overrepresented RNA structural family.

### RT-qPCR experiments

The K562 cell line was the kind gift of C. David Allis and subsequently authenticated by the American Type Culture Collection (ATCC). Four independent replicates of 10 million K562 cells were grown in Roswell Park Memorial Institute 1640 Medium (Gibco), supplemented with 2mM Glutamine and 10% Fetal Bovine Serum. Nuclear fractionation of the four replicates followed the previously published protocol [4], with the addition of an equal amount of the external RNA control consortium (**ERCC**) spike-in RNA standards to the CPE and SNE prior to TRIzol RNA extraction. 1 μg of RNA from each extract was used for reverse transcription with MMLV HP Reverse Transcriptase (Lucigen). A qPCR melt curve analysis was also performed to ensure the specificity of each primer set used in the experiment. Cycle quantification (**Cq**) values from qPCR with PowerUp SYBR Green (Applied Biosystems) were normalized to the ERCC spike-in RNA standard #42 (ΔCq = Cq−*Cq*_spike−in_) per replicate [48]. The normalized ΔCq value was used to calculate abundances (*A* = 2^(−ΔCq)^) of the novel and two control RNAs (**Fig 2F**).

### Other statistics

Other statistical analyses were performed using R (**S2 File**). The coding probability of RNA transcripts was calculated using Coding Potential Calculator 2 (**CPC2**) [22]. AUC was computed using the ROCR (v1.0–7) package [49].

## Supporting information

S1 Fig Prevalence of epigenetic and transcriptional markers in nuclear and total RNA. Transcriptomic (Trans) loci were defined by the presence of ENCODE ChIP-seq peaks or similarity to annotated Rfam families (lncRNA). Peaks of epigenetic marks and transcription factor-occupancy show ChIP-seq results downloaded from ENCODE. Mark of interest (peaks), were compared with each transcriptome and assigned as occurring in both, only one, or neither (at least 1nt, ignoring transcript orientation). The assignment is indicated by the bar color Hallmarks are ordered according to the percentages of peaks overlapping with only the nuclear RNA transcriptome (darkest bar). CPE: Chromatin Pellet Extract; SNE: Soluble Nuclear Extract. **S2 Table** lists the data resources.
(EPS)

S2 Fig Noise filtering and transcript length. **a**, The width distribution of all transcripts built in the four pipelines showing Taco assembles relatively shorter transcripts, with 83% of Taco-assembled RNAs are shorter than 1k bases. **b**, The width distribution of all transcripts built in the four pipelines showing Taco assembles relatively shorter transcripts, with 83% of Taco-assembled RNAs are shorter than 1kbp.
(EPS)

S3 Fig Workflow of categorizing RNA into ‘mRNA’, intergenic RNA, or antisense RNA. (EPS)

S4 Fig Pearson correlations between the identified intergenic RNAs and their neighbor gene. Besides the four types of pairing presented in the Fig 4D in the main manuscript, the dashed line showing a random control which is the correlation between icheRNAs and randomly selected coding genes. The density estimated from this control has been used to calculate the ‘relative density’ of icheRNAs in Fig 4D. The controls estimated for other three types of pairing are not showing for simplification. To pair an intergenic genomic feature with its neighboring gene, the adjacent upstream or downstream gene with the highest magnitude PCC is selected. The kernel density at a certain PCC value is plotted. Two vertical dashed lines mark significant cutoffs of PCC values at -0.8 or 0.8.
(EPS)

S5 Fig Normalized expression values of fractionate RNA classes. Identified chromatin-enriched (top),—depleted (middle), and -independent (bottom) loci were respectively isolated into three subclasses and plotted. Fig 4E in the main manuscript showing the top- and middle-left subpanels for simplicity.
(EPS)

S6 Fig Two groups of cheRNAs with H3K9me3 signal concurs chromatin modification patterns of active enhancers. Average ChIP-seq read density versus input in K562 cells of (**a, d**) H3K9me3 (**b, e**) POL II and H3K4me3 profiles, or H3K27ad and EP300 profiles (c, f) at promoters (±1kb centered at TSS) of two subclasses of cheRNAs compared to unexpressed RNAs (decoded in colors). The cheRNAs overlapping with coding genes on the same strand on top, while antisense to coding genes at the bottom. P-values calculated by two-sided Wilcoxon rank sum test, NS p>0.05, * p<0.01, ** p<1e-10, **** p<2.2e-16.
(EPS)

S7 Fig Fourteen major RNA structural groups in the Rfam database (v13, hg38). **a**, Pi plot showing the proportion in RNA structural motifs per group; **b**, histogram of RNA structural motif widths compared to nuclear RNA-seq transcriptome (Tuxedo-ch). Color decoding the fourteen major RNA structural groups.
(EPS)

S8 Fig Stacked bar chart of asRNAs in three categories by their transcript lengths. The majority (>80%) of those 30 TDF-failed asRNAs were extremely long RNAs (length>50k bases). In contrast, there were approximately 20% extremely long RNAs among the overall asRNAs or among the identified as-cheRNAs.
(EPS)

S1 Table Publicly accessible omics datasets analyzed in this study. (PDF)

S2 Table Genomic landscapes re-analyzed in S1 Fig. (PDF)

S3 Table Novel lncRNA coordinates and their RT-qPCR primers used in this study. (XLSX)

S4 Table Canonical cheRNAs can be better identified by Tuxedo and Concatenating methods. (PDF)

S1 File Source file for the Tuxedo pipeline. (TXT)

S2 File Supplementary Methods. The identifications of Tuxedo-ch in three cell types are accessible on GitHub (https://github.com/xyang2uchicago/Tuxedo-ch).
(DOCX)
