# Supplementary material for: Chromatin-enriched RNAs mark active and repressive cis-regulation: An analysis of nuclear RNA-seq
Source: PLoS Comput Biol. 2020 Feb 10;16(2):e1007119. doi: 10.1371/journal.pcbi.1007119 (PMC7034927; doi:10.1371/journal.pcbi.1007119)
Supplement: S1 Table — (PDF) [file pcbi.1007119.s009.pdf]

S1 Table: Publically accessible omics datasets analyzed in this study.

DATASET_NAME	SEQ TYPE	GENOME	GEO_ SERIES	GEO_ ACCESSION	ENCODE_ ACCESSION	RNA-seq running spots (seq depth)
HEK293_CPE1	RNA-seq	HG38	GSE66478	GSM1623143		67.2M
HEK293_CPE2	RNA-seq	HG38	GSE66478	GSM1623144		98M
HEK293_CPE3	RNA-seq	HG38	GSE66478	GSM1623145		60.5M
HEK293_SNE1	RNA-seq	HG38	GSE66478	GSM1623140		64.6M
HEK293_SNE2	RNA-seq	HG38	GSE66478	GSM1623141		73.4M
HEK293_SNE3	RNA-seq	HG38	GSE66478	GSM1623142		61.4M
H1_CPE1	RNA-seq	HG38	GSE83531	GSM2208157		30.6M
H1_CPE2	RNA-seq	HG38	GSE83531	GSM2208158		27.9M
H1_CPE3	RNA-seq	HG38	GSE83531	GSM2208159		29.8M
H1_SNE1	RNA-seq	HG38	GSE83531	GSM2208160		26.4M
H1_SNE2	RNA-seq	HG38	GSE83531	GSM2208161		31.3M
H1_SNE3	RNA-seq	HG38	GSE83531	GSM2208162		25.9M
K562_CPE1	RNA-seq	HG38	GSE83531	GSM2208147		33.3M
K562_CPE2	RNA-seq	HG38	GSE83531	GSM2208148		31.3M
K562_CPE3	RNA-seq	HG38	GSE83531	GSM2208149		41.4M
K562_SNE1	RNA-seq	HG38	GSE83531	GSM2208150		32M
K562_SNE2	RNA-seq	HG38	GSE83531	GSM2208151		34.2M
K562_SNE3	RNA-seq	HG38	GSE83531	GSM2208152		30.1M
K562_total_RNA-seq_	RNA-seq	HG38	GSE78558	GSM2072364	ENCFF010QAI	78.2M
K562_total_RNA-seq_	RNA-seq	HG38	GSE78558	GSM2072365	ENCFF345SBQ	58.3M
K562_total_RNA-seq_	RNA-seq	HG38	GSE88622	GSM2344296	ENCFF509AOR	39.1M
K562_total_RNA-seq_	RNA-seq	HG38	GSE88622	GSM2344295	ENCFF745GPL	37M
K562_nuclear_polyA_	RNA-seq	HG38	GSE88339	GSM2343814	ENCLB278NDX	44.3M
K562_nuclear_polyA_	RNA-seq	HG38	GSE88339	GSM2343815	ENCLB538THW	47.4M
K562_nuclear_total_	RNA-seq	HG38	GSE87982	GSM2343135	ENCLB873LMQ	24.8M
K562_nuclear_total_	RNA-seq	HG38	GSE87982	GSM2343136	ENCLB645CDM	24.8M
K562 GRO-Seq	GRO-seq	HG19	GSE60454	GSM1480325		
K562_ATAC_1	ATAC-seq	HG19	GSE70482	GSM1782764		
K562_ATAC_2	ATAC-seq	HG19	GSE70482	GSM1782765		
K562_POL2_1	ChIP-seq	HG38	GSE31477	GSM935389	ENCFF730DLS	
K562_POL2_2	ChIP-seq	HG38	GSE31477	GSM935475	ENCFF668VIK	
K562_POL2_3	ChIP-seq	HG38	GSE91721	GSM2423412/	ENCFF285MBX	
K562_POL2_4	ChIP-seq	HG38	GSE32465	GSM803410	ENCFF182YZG	
K562_POL2_5	ChIP-seq	HG38	GSE31477	GSM935473	ENCFF099NYA	
K562_POL2_6	ChIP-seq	HG38	GSE91426		ENCFF881ONC	

K562_H3K27me3_1	ChIP-seq	HG38	GSE29611	GSM733658	ENCFF049HUP
K562_H3K27me3_2	ChIP-seq	HG38	GSE31755	GSM788088	ENCFF031FSF
K562_H3K27ac	ChIP-seq	HG38	GSE29611	GSM733656	ENCFF038DDS
K562_H3K9me3	ChIP-seq	HG38	GSE29611	GSM733776	ENCFF371GMJ
K562_H3K4me3_1	ChIP-seq	HG38	GSE31755	GSM788087	ENCFF961SPZ
K562_H3K4me3_2	ChIP-seq	HG38	GSE29611	GSM733680	ENCFF909PMV
K562_H3K4me3_3	ChIP-seq	HG38	GSE96303		ENCFF616DLO
K562_H3K4me3_4	ChIP-seq	HG38	GSE35583	GSM945165	ENCFF148POZ
K562_H3K4me1	ChIP-seq	HG38	GSE31755	GSM788085	ENCFF159VKJ
K562_EP300	ChIP-seq	HG38	GSE31477	GSM935401	ENCFF755HCK
K562_SUZ12	CHIP-seq	HG38	GSE91742		ENCSR412CTM
K562_EZH2	ChIP-seq	HG38			ENCSR000AQE

While mutiple resource IDs are available, we downloaded it from the ENCODE by ENCODE_ACCESSION IDs.
While only hg19 landscape is available, we liftover its landscape to the hg38 genome.
