# Supplementary material for: Chromatin-enriched RNAs mark active and repressive cis-regulation: An analysis of nuclear RNA-seq
Source: PLoS Comput Biol. 2020 Feb 10;16(2):e1007119. doi: 10.1371/journal.pcbi.1007119 (PMC7034927; doi:10.1371/journal.pcbi.1007119)
Supplement: S2 Table — (PDF) [file pcbi.1007119.s010.pdf]

Table S2: Genomic landscapes re-analyzed in S1 Fig.

Feature type	marker	ID	# of peaks
Histone	ATAC	GSM1782764	65,009,291
	ATAC	GSM1782765	
	EP300	ENCFF755HCK	28,757
	H3K27ac	ENCFF038DDS	52,334
	H3K4me1	ENCFF159VKJ	108,229
	H3K4me2	ENCFF118PIE	66,293
	H3K4me3	ENCFF148POZ	118,763
	H3K4me3	ENCFF616DLO	
	H3K4me3	ENCFF909PMV	
	H3K4me3	ENCFF961SPZ	
	H3K9me3	ENCFF371GMJ	5,584
Transcriptomic	rfam antisense		97
	rfam cisReg		647
	rfam lncRNA		138
	POLR2A	ENCFF099NYA	169,631
	POLR2A	ENCFF182YZG	
	POLR2A	ENCFF285MBX	
	POLR2A	ENCFF668VIK	
	POLR2A	ENCFF730DLS	
	POLR2A	ENCFF881ONC	
TF-binding	ATF2	ENCFF803FHN	46,737
	ATF3	ENCFF467WOR	7,875
	BACH1	ENCFF543FNN	4,707
	BRCA1	ENCFF652NES	815
	BRD4	ENCFF806CQB	8,493
	CBX3	ENCFF951BQB	26,789
	CBX5	ENCFF403TAE	7,022
	CBX8	ENCFF210GJE	4,697
	CEBPB	ENCFF321KQD	71,925
	CEBPB	ENCFF813LOW	
	CTCF	ENCFF119XFJ	200,637
	CTCF	ENCFF396BZQ	
	CTCF	ENCFF519CXF	
	CTCF	ENCFF843VHC	
	E2F6	ENCFF417DTI	51,227
	E2F6	ENCFF533GSH	
	EGR1	ENCFF175VSS	103,204
	EGR1	ENCFF375RDB	
	EGR1	ENCFF561OGS	
	FOSL1	ENCFF087MFG	8,194
	GABPA	ENCFF124HAC	15,818
	GTF2F1	ENCFF478HYJ	5,219
	H2AFZ	ENCFF921IKK	100,908
	H3K9ac	ENCFF306MNO	172,520
	H3K9ac	ENCFF558JOB	

HDAC2	ENCFF363GSV	
HDAC2	ENCFF618YRQ	48,253
HDAC2	ENCFF741IMY	
HDAC6	ENCFF295GBP	1,570
JUN	ENCFF032UMW	
JUN	ENCFF167WUZ	
JUN	ENCFF394CEC	50,782
JUN	ENCFF672LKE	
JUN	ENCFF881AVX	
JUND	ENCFF213EYD	47,477
KDM1A	ENCFF483BRD	80,331
KDM1A	ENCFF796VMI	
KDM5B	ENCFF668XLN	22,315
MAFK	ENCFF893SCL	26,862
MAX	ENCFF618VMC	97,737
MAX	ENCFF900NVQ	
MXI1	ENCFF243QTL	8,988
PHF8	ENCFF952YDR	28,768
RBBP5	ENCFF666PCE	24,374
REST	ENCFF023ZUW	63,662
REST	ENCFF290ESJ	
RING1	ENCFF779XNE	1,077
RNF2	ENCFF349MSP	
RNF2	ENCFF462AZY	69,849
RNF2	ENCFF741CLJ	
RNF2	ENCFF820LKT	
SAP30	ENCFF103RHL	14,223
SIN3A	ENCFF407VGB	15,822
SIN3A	ENCFF802JAN	
SIX5	ENCFF247LOF	3,590
SP1	ENCFF452LDK	14,782
SUZ12	ENCFF856HYC	2,454
TAF1	ENCFF453TIB	19,263
TAF7	ENCFF852NOL	685
TCF12	ENCFF912LXU	45,012
TCF12	ENCFF952JIK	
TEAD4	ENCFF547MLB	36,110
USF1	ENCFF717KGR	21,382
USF2	ENCFF425FVY	3,542
WDR5	ENCFF985TIE	6,630
YY1	ENCFF024TJO	
YY1	ENCFF635XCI	51,788
YY1	ENCFF953BTB	
ZNF143	ENCFF700GZI	29,840
ZNF274	ENCFF323AWS	3,440
ZNF274	ENCFF498VQZ	
