# Supplementary material for: Chromatin-enriched RNAs mark active and repressive cis-regulation: An analysis of nuclear RNA-seq
Source: PLoS Comput Biol. 2020 Feb 10;16(2):e1007119. doi: 10.1371/journal.pcbi.1007119 (PMC7034927; doi:10.1371/journal.pcbi.1007119)
Supplement: S4 Table — (PDF) [file pcbi.1007119.s012.pdf]

**S4 Table. Canonical cheRNAs can be better identified by Tuxedo and Concatenating methods.** Colored cells are validated in  tissue-specific experiments and used as gold standards.

| **16 alidations** | | | | HEK293 | | | | K562 | | | |
|---|---|---|---|---|---|---|---|---|---|---|---|
| **result** | **cell** | **method** | **RNA Symbol** | Werner | Tuxedo | Concat. | Taco | Werner | Tuxedo | Concat. | Taco |
| **CPE** | **HEK293** | RT-PCR | KCNQ1OT1 | CPE | CPE | CPE | None | | | | |
| | **HEK293** | RT-PCR | XIST | HI-DATA | CPE | CPE | CPE | | | | |
| | **HEK293 /K562** | RT-PCR | PVT1 | - | CPE | CPE | - | CPE | CPE | CPE | CPE |
| | **K562** | function | BONIFACIO | | | | | CPE | CPE | CPE | CPE |
| | **K562** | function | ILYICH | | | | | CPE | CPE | CPE | CPE |
| | **K562** | function | HIDALGO | | | | | - | CPE | CPE | CPE |
| **SNE** | **HEK293** | RT-PCR | GAPDH | SNE | SNE | SNE | SNE | | | | |
| | **HEK293** | RT-PCR | ACTB | SNE | SNE | SNE | SNE | | | | |
| | **HEK293 /K562** | RT-PCR | MYC | SNE | SNE | SNE | SNE | SNE | SNE | SNE | SNE |
| | **K562** | RNA-seq reads | B3GNT2 | | | | | SNE | SNE | SNE | SNE |
| | **K562** | RNA-seq reads | IL6 | | | | | - | - | - | NA |
| | **K562** | RNA-seq reads | PDCD6IP | | | | | - | SNE | SNE | SNE |
| **intermediate** | **HEK293** | RT-PCR | HOTAIR | SNE | - | - | NonA | | | | |
| | **HEK293** | RT-PCR | DLX6-AS1, Evf-2_5p | - | CPE | CPE | NonA | | | | |
| **correct prediction:** | | | | 5 | 7 | 7 | 4 | 5 | 7 | 7 | 7 |
| **number of gold standarad:** | | | | 8 | 8 | 8 | 8 | 8 | 8 | 8 | 8 |
| **PPV** | | | | 0.63 | 0.88 | 0.88 | 0.50 | 0.63 | 0.88 | 0.88 | 0.88 |

**NonA:** not assembled
**NA**: not tested because of low expression level
**HIDATA**: not tested by Cuffdiff because of high expression level
**"-"**: tested but not significantly differentially expressed
**PPV:** number of correct predictions /number of gold standard in each cell line (8 gold standard in HEK293 while 8 gold standard in K562)
