# Supplementary material for: Chromatin-enriched RNAs mark active and repressive cis-regulation: An analysis of nuclear RNA-seq
Source: PLoS Comput Biol. 2020 Feb 10;16(2):e1007119. doi: 10.1371/journal.pcbi.1007119 (PMC7034927; doi:10.1371/journal.pcbi.1007119)
Supplement: S2 File — The identifications of Tuxedo-ch in three cell types are accessible on GitHub (https://github.com/xyang2uchicago/Tuxedo-ch). (DOCX) [file pcbi.1007119.s014.docx]

## **Supplementary Method for**

**Chromatin-enriched RNAs mark active and repressive cis-regulators: an analysis of nuclear RNA-seq**

Xiangying Sun^2,1^, Zhezhen Wang^1^, Johnathon M Hall^3^, Carlos Perez-Cervantes^1^, Alexander J Ruthenburg^3^, Ivan P Moskowitz^1,3^, Michael Gribskov^2,4^, Xinan H Yang^1,^*

1. Department of Pediatrics, The University of Chicago, Chicago, IL 60637, USA

2. Department of Biological Sciences, Purdue University, West Lafayette, IN 47906, USA

3. Department of Molecular Genetics and Cell Biology, The University of Chicago,

4. Department of Computer Science, Purdue University, West Lafayette, IN 47906, USA

**Datasets**

RNA-seq raw datasets (in HEK293, H1 and K562 cell lines) were obtained from the NCBI Short-Read Archive (SRA) (**S1 Table**). For the K562 cells, nuclear RNA sequencing, the ChIP-seq of multiple histone marks and transcription factors and ATAC-seq datasets were downloaded from ENCODE data portal [1] (https://www.encodeproject.org/) (**S1 Table, S2 Table**). The noncoding RNA family were defined by Rfam (v13) [2].

### **Identification of chromatin-enriched RNA (cheRNA) using four pipelines**

We compared four pipelines, namely Werner, Concatenating, Tuxedo-ch, and Taco, for the identification of chromatin enriched RNAs (**Fig 1c**). Werner was executed by strictly following the analysis steps published in Werner’s paper [3]. Briefly, there are four steps in each pipeline: sequence mapping, transcript assembly, transcriptome construction, and signature identification.

The sequence mapping step is the same in all four pipelines. First, the reads were mapped against the human genome version GRCh38.p10 using Tophat (v2.1.1) [4] with default parameters for stranded RNA-seq libraries (e.g., tophat -p 8 --library-type=fr-firststrand -G gencode.v25.gtf GRCH38.genome -o CPE1 CPE1.fastq).

Strategies used in transcript assembly varied in four different pipelines. In the Werner pipeline, de novo assembly was applied only on the three biological Chromatin Pellet Extract (**CPE**) replicates using Cufflinks (v2.2.1) [5] (e.g., cufflinks -p 8 -u -N -library-type fr-firststrand -o cufflinks_CPE1 CPE1.bam), while reference-guided assembly (e.g., cufflinks -p 8 -u -N -library-type fr-firststrand -G gencode.v25.gtf) were applied on the three biological Soluble Nuclear Extract (**SNE**) replicates. In Concatenatin, Tuxedo-ch and Taco pipelines, we independently applied the reference annotation-based transcript (**RABT**) assembly, which assembles both known and novel transcript. Specifically, we run the RABT assembly on the three biological CPE replicates and three biological SNE replicates by Cufflinks [5] using “cufflinks -g” option with GENCODE (v25) annotation as reference (e.g., cufflinks -u -N -library-type fr-firststrand -g gencode.v25.gtf -o cufflinks_CPE1 CPE1.bam).

Strategies used to construct transcriptome also varied in four different pipelines. In Werner, we did:

1. CPE replicates and SNE replicates were separately combined using Cuffmerge (v2.2.1) [6], resulting in both CPE transcriptome (e.g., cuffmerge -p 8 -o CPE_cuffmerge CPE_transcripts.txt) and SNE transcriptome (e.g., cuffmerge -p 8 -o SNE_cuffmerge SNE_transcripts.txt).
2. Reused transcript identifiers in the CPE transcriptome ‘XLOC_’ were renamed to ‘CLOC_’ to differentiate them from the transcript in the SNE transcriptome.
3. Only transcripts longer than 1000bp were kept in CPE transcriptome. – Note that this sub-step is specific to the Werner pipeline according to the author which may cause a bias to longer transcripts [3]
4. Two .bed files of CPE transcriptomes (‘CLOC_’) and SNE transcriptomes (‘XLOC_’) were obtained from their respective Cufflinks output .gtf files using gtf2bed in BEDOPS (v2.4.28) [7].
5. Next, we retrieved CPE-unique transcriptome using intersectBed (e.g., intersectBed -s -v -a CLOC.bed -b XLOC.bed) in bedtools (v2.26.0) [8].
6. These CPE-unique transcriptomes were then combined with SNE transcriptomes using ‘cat’ command to build the transcriptome for differential expression analysis.

The other three pipelines were similar to Werner except the sub-steps 1) and 3). In Concatenating, we used Cuffmerge to merge three CPE replicates (e.g., cuffmerge -p 8 -o CPE_cuffmerge CPE_transcripts.txt) and three SNE replicates (e.g., cuffmerge -p 8 -o SNE_cuffmerge SNE_transcripts.txt) separately to get the CPE transcriptome and SNE transcriptome.

In Tuxedo-ch, we used Cuffmerge to merge all CPE and SNE replicates together to make an annotation for differential expression analysis (e.g., cuffmerge -p 8 -o tuxedo_cuffmerge ALL_transcripts.txt).

And in Taco, we used Taco [9] to merge all CPE and SNE replicates together to build the transcriptome for differential expression analysis.

In the last step to identify cheRNA signatures, Werner used Cuffdiff (v2.2.1) [6] with standard options (cuffdiff -p 8 -o cuffdiff.out --library-type fr-firststrand -L SNE,CPE -u combined_transcriptome.gtf K562_SNE1.bam, K562_SNE2.bam, K562_SNE3.bam K562_CPE1.bam, K562_CPE2.bam, K562_CPE3.bam). As a result, un-transcribed RNAs were identified as RNAs with a “NOTEST” value under “Test status” column in “gene_exp.diff” table. When contrasting expression levels in CPE samples to SNE samples, RNAs with FoldChange>1 and q_value<0.05 were identified as CPE-enriched RNA (**cheRNAs**) and RNAs with FoldChange<1 and q_value<0.05 were identified Soluble Nuclear-Extracted RNAs (**sneRNAs**).

In Concatenating, Tuxedo-ch and Taco pipelines, we applied the same advanced computational strategy (limma) which generally showed higher precision and shortest runtimes than cuffdiff in RNA-seq data analysis [10]. Specifically, we did:

1. Used HTSeq (v.0.7.0) [11] to get the raw counts of transcripts (e.g., htseq-count -f bam -s no -m intersection-nonempty CPE1.bam tuxedo_transcriptome.gtf > CPE1_geneCounts.out).
2. Transformed the expression of RNAs from raw counts to counts per million (**CPM**). RNAs with CPM<1 are considered as un-transcribed. Only RNAs expressed in at least 3 out of 6 samples were retained for further analysis.
3. Normalization of RNA expression was performed by the method of trimmed mean of M-values (**TMM**).
4. Used limma package in R [12] to do differential expression analysis comparing CPE samples with SNE samples, per cell type. The expected FDR was estimated using the Benjamini-and-Hochberg method.
5. Transcripts having FDR<0.05 and FoldChange>1.2 were identified as chromatin enriched RNAs and transcripts having FDR<0.05 and FoldChange<0.83 were identified as chromatin depleted RNAs.

### **Discussion on the length of all assembled transcripts (S2 Fig).**

Approximately half of the assembled transcripts had lengths between 200-1000 bases and show similar log-normal distributions for Tuxedo-ch and Concatenating (Fig S2a). In contrast, transcripts assembled by Werner were generally longer (71% of the assembled transcripts are longer than 1000 bases, Fig S2b); while transcripts assembled by Taco were much shorter (83% of assembled transcripts are shorter than 1000 bases). The TACO assembler employs an algorithm based on change-point detection via binary segmentation to predict transcript structure [9]. This algorithm is more robust in the assembly of annotated and conserved transcript such as mRNA. However, when it was applied to assembly of noncoding RNA, the TACO assembler overestimated the degree of alternative splicing and results in a large number of truncated transcripts. This is incorrect since only a small fraction of lncRNA undergo splicing [13].

### **Calculating numbers of coordinate-overlaps**

The numbers of coordinate-overlapped transcripts are calculated by using the R package ChIPpeakAnno [14, 15] with the “findOverlapsOfPeaks” function. Transcripts with a coordinate-overlapping of 1bp or more on the same strand are considered to be overlapped. If one transcript in one set is (or multiple transcripts are) overlapped with multiple transcripts in the other set, the number of overlapped transcripts is counted as the minimal number of involved transcripts in any of the two groups. The venn diagrams shown in **Figure 2 a-c** are plotted using the R package ChIPpeakAnno with the “makeVennDiagram”.

### **Calculating proportions of transcripts coincident with GRO-seq/POL II signals**

The K562 POLL II “bed narrowPeak” files in GRCh38 are downloaded from ENCODE. GRO-seq “bigwig” files in hg19 are downloaded from GEO [16] and a liftover of the hg19 annotations to GRCh38.p10 were then generated using an online tool called Batch Coordinate Conversion (liftOver) in UCSC genome browser [17]. Transcripts overlapped 1bp or more with GRO-seq/POL II peaks by coordinates are defined as transcripts coincident with GRO-seq/POL II signals. Overlapping between transcripts and GRO-seq/POL II peak regions is done by using the R package GenomicRanges [18] with the “findoverlaps” function.

### **Categorizing transcripts into mRNA, intergenic RNA (iRNA), and antisense RNA (as-RNA) (S3 Figure)**

We categorized the assembled RNAs into three subgroups based on their relative genomic locations to GENCODE (v25)-annotated protein-coding genes. We firstly overlapped the coordinates of all assembled RNAs with GENCODE annotated protein-coding genes by using the “findOverlaps” function in R package GenomicRanges (v1.32.3) [18]. Those assembled-RNAs that were not overlapped with any protein-coding genes were categorized as intergenic RNAs (iRNAs). The RNAs overlapping with protein-coding genes on the same strand were spitted into two sub-groups: those with an overlapped region accounts for at least 50% of the assembled RNA region were categorized as ‘mRNAs’; and the others were categorized as iRNAs. Finally, the assembled RNAs whose coordinates overlapped with protein-coding genes on the opposite strand were identified as antisense RNAs. Among those antisense RNAs, the ones that overlapped with protein-coding promoters (1000 bp windows around TSS of genes) were further categorized as antisense RNAs at 5UTR; other antisense RNAs were then categorized as antisense RNAs at 3UTR.

### **Coding probability calculation**

The coding probability of RNA transcripts was calculated using Coding Potential Calculator 2 (**CPC2**) [19]. CPC2 assessed coding probability by employing a support vector machine model based on four sequence intrinsic features: Fickett TESTCODE score of DNA sequences [20], open reading frame (**ORF**) length, ORF integrity, and isoelectric point.

### **AUC analysis**

AUC analysis was performed using the ROCR (v1.0-7) package in R [21]. The commonly identified 731 cheRNAs or 3573 sneRNAs by all four pipelines were used as a gold standard to calculate the accuracy of prediction in AUC analysis.

### **Chromatin states analysis and comparison**

When comparing chromatin states of interesting loci, we used ChIP-seq signals directly from BAM files instead of the published peak files for better sensitivity. Files meeting the following criteria were included in the analysis: (1). Format = Bam; (2) Genome version = GRCh38; (3). Output type = alignments.

To compare different chromatin features and chromatin accessibility, the metagene analysis was performed at either body regions or promoter regions (±1kb of TSS) of RNAs using the Bioconductor package metagene (v2.14.0) [22]. When comparing ChIP-Seq signals using the downloaded bam files (which may ignore the ChIP-seq input control) with metagene analyses, we input not only the bam file for a histone mark but also its input control. Briefly, three steps were performed for meta-gene analysis:

1) The read coverages of all selected regions were extracted from BAM files and normalized to reads per million aligned (**RPM**) using the Bioconductor package metagene.

2) We divided each interested region into 100 equally-sized bins, and calculated the averaged RPM within each bin.

3) Metagene profiles were plotted in the format of a ribbon plot or a box plot. If plotted in a ribbon plot, lines represent averaged RPM and ribbons represent the 95% confidence interval of the mean calculated using 1000 bootstraps; If plotted in a box plot, each box represents the distribution of averaged RPM at each bin.

4) To statistically compare two averaged RPM distributions, two-sided Wilcoxon rank sum test was performed to calculate the p-value.

### **Retrieving ChromHMM predicted enhancer-driven RNAs (eRNAs)**

To retrieve ChromHMM predicted eRNAs in K562 cell line, we downloaded the broad Chromatin State Segmentation by Hidden Markov Model from ENCODE (Broad **ChromHMM**) [23] profile in hg19 for K562 cell line from ENCODE (http://genome.ucsc.edu/encode/downloads.html). A map of these downloaded hg19 annotations to GRCh38.p10 was then conducted using an online tool called Batch Coordinate Conversion (liftOver) in the UCSC genome browser [17]. In this work, ChromHMM-predicted eRNAs were defined as intergenic RNAs overlap (at least 1bp) with ChromHMM-predicted “Strong enhancer” regions.

### **Retrieving FANTOM profiles**

To extract FANTOM-predicted eRNAs in the K562 cell line, we downloaded the FANTOM-predicted enhancer regions in hg19 (ubiquitous_enhancers_cells.bed.txt) from FANTOM5 consortium (<http://slidebase.binf.ku.dk/human_enhancers/presets)> [24]. A liftover of the hg19 annotations to GRCh38.p10 for the downloaded profile were then generated using an online tool called Batch Coordinate Conversion (liftOver) in the UCSC genome browser [17]. FANTOM-predicted eRNAs were defined as intergenic RNAs overlap (at least 1bp) with FANTOM-predicted enhance regions.

### **PolyA RNA-seq and total RNA-seq analysis (Figure 4e and S5 Figure)**

To compare expression levels of nuclear RNAs in different RNAseq libraries, we downloaded the raw sequencing datasets of K562 nuclear polyA RNA-seq (GSE88339) and nuclear total RNA-seq (GSE87982) from ENCODE data portal (**S1 Table**). Reads were aligned to the human genome version GRCh38.p10 using Tophat (v2.1.1) [4] (e.g., tophat -p 8 --library-type=fr-firststrand -G gencode.v25.gtf GRCH38.genome -o polyA1 polyA1.fastq). Then the Fragments Per Kilobase Million (**FPKM)** of RNA transcripts were calculated using Cufflinks (v2.2.1) (e.g., cufflinks -p 8 -u -N -library-type fr-firststrand -o FPKM_polyA1 -G gtf polyA1.bam). When making dot plots in **Figure 4e and S5 Figure**, only expressed RNAs (with CPM>1) were plotted.

### **ChIP-seq peak signal**

ChIP-seq peak signals were downloaded from ENCODE as “bed narrowPeak” files (**S1 Table**). When one sample includes several replicates, we used the “bed narrowPeak” file with the Irreproducible Discovery Rate (IDR) values thresholded at the optimization precision (“optimal idr thresholded peaks”). When multiple samples are available and collected for one mark, we generated a union signal which was the pool of ChIP-seq peaks identified at least once from biological replicates. All files were downloaded with GRCh38 mapping assembly.

These bed/wig files generated from ENCODE used a score associated with each peak (enriched interval) which is the mean signal value across the interval. (Note that a broad region with moderate enrichment may deviate from the background more significantly than a short region with high signal.) The input control information is on the same page where the bed/wig/bam file is download.

### **Calculating proportions of transcripts overlapping with LADs**

The genomic coordinates of human (hg19) fibroblast LADs are downloaded from ENCODE (http://compbio.med.harvard.edu/modencode/webpage/lad/human.fibroblast.DamID.hg19.bed) and a liftover of the hg19 annotations to GRCh38.p10 were then generated using an online tool called Batch Coordinate Conversion (liftOver) in UCSC genome browser [17]. Transcripts embedded in LADs are defined if more than 50% of the transcript overlaps with genomic coordinates of LADs. Overlapping between genomic coordinates of transcripts and LADs is done by using the R package GenomicRanges [18] with the “findoverlaps” function.

### **Calculating proportions of transcripts overlapping with class 1 TEs**

The annotation of class 1 TEs in human (GRCh38.p10) is downloaded from RepeatMasker (<http://www.repeatmasker.org/species/hg.html>) [25]. Transcripts overlapping with class 1 TEs are defined if the sequence of the transcript contains at least one sequence of class 1 TEs.

### **RNA:DNA:DNA triplex prediction**

RNA:DNA:DNA triple-helix formations (triplex) is one mechanism that associates lncRNA to repressive chromatin [26]. To explore molecular mechanisms behind the observed gene downregulation at as-cheRNAs, we scanned the possibility of triple-helix formations among these 756 asRNAs expressed in K562 cells. We employed the Triplex Domain Finder (**TDF**) analysis that inputs the FASTA file of each asRNA and the symbol of its local gene on the opposite strand [27]. The ‘TDF-promoter test’ estimated the possibility of each asRNA to form triple helice(s) with the promoter of its local gene compared to that with all human gene promoters [27]. A TDF-promoter predicted P-value <0.05 was considered as possible triple-helix formation between that asRNA and local gene. Then, Fisher's exact test was performed to test the null hypothesis of independence between chromatin enrichment and triple-helixes formation. This tests were respectively run using two Linux machine: Midway2 with a wall-time of 18 hours and memory limit of 58G, Gardner with a wall-time of 50 hours and memory limit of 128G (See GitHub (<https://github.com/xyang2uchicago/Tuxedo-ch> for details). There were 30 asRNAs running out of time wall or memory. These 30 asRNAs were excluded from the final Fisher's exact test (**Fig 6f**).

## **Reference for Supplementary Materials and method**

1. Sloan CA, Chan ET, Davidson JM, Malladi VS, Strattan JS, Hitz BC, et al. ENCODE data at the ENCODE portal. Nucleic Acids Res. 2016;44(D1):D726-32. doi: 10.1093/nar/gkv1160. PubMed PMID: 26527727; PubMed Central PMCID: PMCPMC4702836.

2. Kalvari I, Argasinska J, Quinones-Olvera N, Nawrocki EP, Rivas E, Eddy SR, et al. Rfam 13.0: shifting to a genome-centric resource for non-coding RNA families. Nucleic Acids Res. 2018;46(D1):D335-D42. doi: 10.1093/nar/gkx1038. PubMed PMID: 29112718; PubMed Central PMCID: PMCPMC5753348.

3. Werner MS, Ruthenburg AJ. Nuclear Fractionation Reveals Thousands of Chromatin-Tethered Noncoding RNAs Adjacent to Active Genes. Cell Rep. 2015;12(7):1089-98. doi: 10.1016/j.celrep.2015.07.033. PubMed PMID: 26257179; PubMed Central PMCID: PMCPMC5697714.

4. Kim D, Pertea G, Trapnell C, Pimentel H, Kelley R, Salzberg SL. TopHat2: accurate alignment of transcriptomes in the presence of insertions, deletions and gene fusions. Genome Biol. 2013;14(4):R36. Epub 2013/04/27. doi: 10.1186/gb-2013-14-4-r36. PubMed PMID: 23618408.

5. Trapnell C, Roberts A, Goff L, Pertea G, Kim D, Kelley DR, et al. Differential gene and transcript expression analysis of RNA-seq experiments with TopHat and Cufflinks. Nat Protoc. 2012;7(3):562-78. doi: 10.1038/nprot.2012.016. PubMed PMID: 22383036; PubMed Central PMCID: PMC3334321.

6. Trapnell C, Williams BA, Pertea G, Mortazavi A, Kwan G, van Baren MJ, et al. Transcript assembly and quantification by RNA-Seq reveals unannotated transcripts and isoform switching during cell differentiation. Nat Biotechnol. 2010;28(5):511-5. Epub 2010/05/04. doi: 10.1038/nbt.1621. PubMed PMID: 20436464; PubMed Central PMCID: PMC3146043.

7. Neph S, Kuehn MS, Reynolds AP, Haugen E, Thurman RE, Johnson AK, et al. BEDOPS: high-performance genomic feature operations. Bioinformatics. 2012;28(14):1919-20. doi: 10.1093/bioinformatics/bts277. PubMed PMID: 22576172; PubMed Central PMCID: PMC3389768.

8. Quinlan AR, Hall IM. BEDTools: a flexible suite of utilities for comparing genomic features. Bioinformatics. 2010;26(6):841-2. Epub 2010/01/30. doi: 10.1093/bioinformatics/btq033. PubMed PMID: 20110278; PubMed Central PMCID: PMC2832824.

9. Niknafs YS, Pandian B, Iyer HK, Chinnaiyan AM, Iyer MK. TACO produces robust multisample transcriptome assemblies from RNA-seq. Nat Methods. 2017;14(1):68-70. doi: 10.1038/nmeth.4078. PubMed PMID: 27869815; PubMed Central PMCID: PMCPMC5199618.

10. Seyednasrollah F, Laiho A, Elo LL. Comparison of software packages for detecting differential expression in RNA-seq studies. Brief Bioinform. 2015;16(1):59-70. doi: 10.1093/bib/bbt086. PubMed PMID: 24300110; PubMed Central PMCID: PMCPMC4293378.

11. Anders S, Pyl PT, Huber W. HTSeq--a Python framework to work with high-throughput sequencing data. Bioinformatics. 2015;31(2):166-9. doi: 10.1093/bioinformatics/btu638. PubMed PMID: 25260700; PubMed Central PMCID: PMCPMC4287950.

12. Ritchie ME, Phipson B, Wu D, Hu Y, Law CW, Shi W, et al. limma powers differential expression analyses for RNA-sequencing and microarray studies. Nucleic Acids Res. 2015;43(7):e47. doi: 10.1093/nar/gkv007. PubMed PMID: 25605792; PubMed Central PMCID: PMCPMC4402510.

13. Tilgner H, Knowles DG, Johnson R, Davis CA, Chakrabortty S, Djebali S, et al. Deep sequencing of subcellular RNA fractions shows splicing to be predominantly co-transcriptional in the human genome but inefficient for lncRNAs. Genome Res. 2012;22(9):1616-25. doi: 10.1101/gr.134445.111. PubMed PMID: 22955974; PubMed Central PMCID: PMCPMC3431479.

14. Zhu LJ, Gazin C, Lawson ND, Pages H, Lin SM, Lapointe DS, et al. ChIPpeakAnno: a Bioconductor package to annotate ChIP-seq and ChIP-chip data. BMC Bioinformatics. 2010;11:237. doi: 10.1186/1471-2105-11-237. PubMed PMID: 20459804; PubMed Central PMCID: PMC3098059.

15. Zhu LJ. Integrative analysis of ChIP-chip and ChIP-seq dataset. Methods Mol Biol. 2013;1067:105-24. doi: 10.1007/978-1-62703-607-8_8. PubMed PMID: 23975789.

16. Edgar R, Domrachev M, Lash AE. Gene Expression Omnibus: NCBI gene expression and hybridization array data repository. Nucleic Acids Res. 2002;30(1):207-10. Epub 2001/12/26. PubMed PMID: 11752295.

17. Kent WJ, Sugnet CW, Furey TS, Roskin KM, Pringle TH, Zahler AM, et al. The human genome browser at UCSC. Genome Res. 2002;12(6):996-1006. doi: 10.1101/gr.229102. PubMed PMID: 12045153; PubMed Central PMCID: PMCPMC186604.

18. Lawrence M, Huber W, Pages H, Aboyoun P, Carlson M, Gentleman R, et al. Software for computing and annotating genomic ranges. PLoS Comput Biol. 2013;9(8):e1003118. doi: 10.1371/journal.pcbi.1003118. PubMed PMID: 23950696; PubMed Central PMCID: PMC3738458.

19. Kang YJ, Yang DC, Kong L, Hou M, Meng YQ, Wei L, et al. CPC2: a fast and accurate coding potential calculator based on sequence intrinsic features. Nucleic Acids Res. 2017;45(W1):W12-W6. doi: 10.1093/nar/gkx428. PubMed PMID: 28521017; PubMed Central PMCID: PMCPMC5793834.

20. Fickett JW. Recognition of protein coding regions in DNA sequences. Nucleic Acids Res. 1982;10(17):5303-18. PubMed PMID: 7145702; PubMed Central PMCID: PMCPMC320873.

21. Sing T, Sander O, Beerenwinkel N, Lengauer T. ROCR: visualizing classifier performance in R. Bioinformatics. 2005;21(20):3940-1. doi: 10.1093/bioinformatics/bti623. PubMed PMID: 16096348.

22. Noguchi H, Park J, Takagi T. MetaGene: prokaryotic gene finding from environmental genome shotgun sequences. Nucleic Acids Res. 2006;34(19):5623-30. doi: 10.1093/nar/gkl723. PubMed PMID: 17028096; PubMed Central PMCID: PMCPMC1636498.

23. Ernst J, Kellis M. ChromHMM: automating chromatin-state discovery and characterization. Nat Methods. 2012;9(3):215-6. doi: 10.1038/nmeth.1906. PubMed PMID: 22373907; PubMed Central PMCID: PMCPMC3577932.

24. Andersson R, Gebhard C, Miguel-Escalada I, Hoof I, Bornholdt J, Boyd M, et al. An atlas of active enhancers across human cell types and tissues. Nature. 2014;507(7493):455-61. doi: 10.1038/nature12787. PubMed PMID: 24670763.

25. Huda A, Jordan IK. Analysis of transposable element sequences using CENSOR and RepeatMasker. Methods Mol Biol. 2009;537:323-36. doi: 10.1007/978-1-59745-251-9_16. PubMed PMID: 19378152.

26. Mondal T, Subhash S, Vaid R, Enroth S, Uday S, Reinius B, et al. MEG3 long noncoding RNA regulates the TGF-beta pathway genes through formation of RNA-DNA triplex structures. Nat Commun. 2015;6:7743. doi: 10.1038/ncomms8743. PubMed PMID: 26205790; PubMed Central PMCID: PMCPMC4525211.

27. Kuo CC, Hanzelmann S, Senturk Cetin N, Frank S, Zajzon B, Derks JP, et al. Detection of RNA-DNA binding sites in long noncoding RNAs. Nucleic Acids Res. 2019;47(6):e32. doi: 10.1093/nar/gkz037. PubMed PMID: 30698727; PubMed Central PMCID: PMCPMC6451187.
